# Characterization of the Bacterial Communities of Life Stages of Free Living Lone Star Ticks (*Amblyomma americanum*)

**DOI:** 10.1371/journal.pone.0102130

**Published:** 2014-07-23

**Authors:** Amanda Jo Williams-Newkirk, Lori A. Rowe, Tonya R. Mixson-Hayden, Gregory A. Dasch

**Affiliations:** 1 Department of Environmental Sciences, College of Arts and Sciences, Emory University, Atlanta, Georgia, United States of America; 2 Graduate Program in Population Biology, Ecology and Evolution, Emory University, Atlanta, Georgia, United States of America; 3 Rickettsial Zoonoses Branch, Division of Vector-Borne Diseases, Centers for Disease Control and Prevention, Atlanta, Georgia, United States of America; 4 Biotechnology Core Facility Branch, National Center for Emerging Zoonotic Diseases, Centers for Disease Control and Prevention, Atlanta, Georgia, United States of America; 5 Laboratory Branch, Division of Viral Hepatitis, Centers for Disease Control and Prevention, Atlanta, Georgia, United States of America; University of Minnesota, United States of America

## Abstract

The lone star tick (*Amblyomma americanum*) is an abundant and aggressive biter of humans, domestic animals, and wildlife in the southeastern-central USA and an important vector of several known and suspected zoonotic bacterial pathogens. However, the biological drivers of bacterial community variation in this tick are still poorly defined. Knowing the community context in which tick-borne bacterial pathogens exist and evolve is required to fully understand the ecology and immunobiology of the ticks and to design effective public health and veterinary interventions. We performed a metagenomic survey of the bacterial communities of questing *A. americanum* and tested 131 individuals (66 nymphs, 24 males, and 41 females) from five sites in three states. Pyrosequencing was performed with barcoded eubacterial primers targeting variable 16S rRNA gene regions 5–3. The bacterial communities were dominated by *Rickettsia* (likely *R. amblyommii*) and an obligate *Coxiella* symbiont, together accounting for 6.7–100% of sequences per tick. DNAs from *Midichloria*, *Borrelia*, *Wolbachia*, *Ehrlichia*, *Pseudomonas*, or unidentified Bacillales, Enterobacteriaceae, or Rhizobiales groups were also detected frequently. *Wolbachia* and *Midichloria* significantly co-occurred in Georgia (*p*<0.00001), but not in other states. The significance of the *Midichloria*-*Wolbachia* co-occurrence is unknown. Among ticks collected in Georgia, nymphs differed from adults in both the composition (*p* = 0.002) and structure (*p = *0.002) of their bacterial communities. Adults differed only in their community structure (*p* = 0.002) with males containing more *Rickettsia* and females containing more *Coxiella*. Comparisons among adult ticks collected in New York and North Carolina supported the findings from the Georgia collection despite differences in geography, collection date, and sample handling, implying that the differences detected are consistent attributes. The data also suggest that some members of the bacterial community change during the tick life cycle and that some sex-specific attributes may be detectable in nymphs.

## Introduction

Ticks transmit a greater diversity of pathogens to humans and domestic animals than any other vector group [1], and the lone star tick (*Amblyomma americanum*) is the most common human-biting tick in the southeastern United States [2]. During the last century *A. americanum* has expanded its range into the northeastern and mid-western states [3]–[6], further increasing the number of people and domestic animals exposed to the tick and the pathogens it transmits.

*Amblyomma americanum* is a three-host tick that is nonspecific in its host use in the immature (larval and nymphal) life stages and has a preference for white-tailed deer (*Odocoileus virginianus*) as adults (reviewed in [6]). As many pathogens are acquired by ticks from their vertebrate hosts, the lack of host specificity throughout much of its life perfectly positions this tick as a vector of multiple zoonotic diseases, including ehrlichioses [7], rickettsioses, tularemia, and perhaps even Southern Tick-Associated Rash Illness (reviewed in [6]). Other bacteria of undefined pathogenicity (*Candidatus* “Midichloria mitochondrii” [8], [9], *Borrelia lonestari* [10]) and several viruses (Lone Star virus [11], Heartland virus [12]) are also associated with this species. Larvae, nymphs, and adult females feed once from a single host before becoming quiescent in the leaf litter and either molting into the next life stage (immatures) or ovipositing (adult females). Adult males take multiple smaller blood meals while seeking feeding females [13].

Tick-borne disease ecology motivates many tick microbiological studies, which frequently use specific assays to survey for the presence of known vertebrate pathogens. Yet many vertebrate pathogens are neither prevalent in the vector population (e.g. [14]) nor abundant within pathogen-infected ticks (e.g. [15]) and may represent only a minority population within the tick microbiome. This suggests that the interactions between vertebrate pathogens and other more common and abundant bacteria found in ticks may be important to the abundance and distribution of pathogen-infected ticks in the environment. Several previous surveys of the bacterial communities of *A. americanum* have provided useful inventories of community members. Sanger sequencing by Clay et al. [16] and Heise et al. [17] and pyrosequencing by Ponnusamy et al. [18] and Yuan [19] of bacterial 16S rRNA gene fragments from *A. americanum* confirmed the common presence of a *Coxiella* that is a likely obligate symbiont [20], [21]. They also detected the low pathogenicity spotted fever group *Rickettsia Candidatus* “Rickettsia amblyommii” (hereafter *R. amblyommii*) as an abundant and common bacterial community member. Additional *Rickettsia* species [17] and a novel *Arsenophonus* were also identified [16], [19], as well as a number of diverse taxa frequently found both environmentally and as animal associates, most commonly *Pseudomonas* [16], [17], [19], Enterobacteracea [16], [17], and Bacillaceae [17], [19].

While the *A. americanum* bacterial community has been well documented, the nature and basis for intraspecific differences are less well explored. Menchaca et al. [22] used semi-conductor sequencing to describe changes in the bacterial communities of laboratory colony derived nymphs and adults to test the effects of engorgement, molting, age, and environmental conditions on bacterial communities. They found an interesting trend towards reduction in community diversity with tick age and environmental stressors under extremely controlled conditions. When pyrosequencing bacterial 16S amplicons from a small sample of 12 *A. americanum*, Ponnusamy et al. found more unique bacterial taxa in males and nymphs than in females. In other ixodid tick species, tick life stage and sex have been used to explain variation in whole bacterial communities [23], [24] and focal bacterial taxa [25]–[27], although the mechanisms behind these differences remain unclear.

Here we characterize the life stage and sex specific bacterial communities of wild caught *A. americanum* collected at five sites in three states using pyrosequencing of variable regions 5–3 of the bacterial *rrs* (16S rRNA) gene. Samples of nymphs and adults from two sites in Georgia were compared to detect differences in community composition and structure between life stages and sexes from a single geographic area. Archived DNAs from adult *A. americanum* were also compared to see if sex-specific bacterial communities were a general phenomenon in this species or a region-specific occurrence. The geographical distribution of our sample sites in combination with our larger sample sizes enabled the identification of significant life stage and sex specific patterns of bacterial community composition and structure that have not been reported from previous studies.

## Methods

### Ethics statement

Tick collection in Georgia was performed in state parks with permission from the Georgia Department of Natural Resources (permits #29-WBH-10-135 and #29-WBH-11-49) and park management. Ticks from other states were collected as described in Mixson et al. [14].

### Tick collection and DNA extraction

All *A. americanum* ticks were collected by running a 1 m^2^ flannel cloth over vegetation. The primary tick DNA sample set (hereafter referred to as the Georgia collection) was collected in 2010–2011 from two ecologically similar forested state parks very near to Atlanta, Georgia (Table 1) and stored in 70% ethanol at 4°C. DNA extraction was performed as described in Bermúdez et al. [28] with the Promega (Madison, WI, USA) Wizard SV 96 Genomic DNA Purification System. Ticks were treated with sequential pre-extraction surface washes of 10% bleach, 70% ethanol, and distilled water to reduce surface contamination. DNAs were stored at 4°C until used. Tick DNAs from our archives (hereafter referred to as the archival collection) were obtained from ticks from three sites on barrier islands in New York (1 site) and North Carolina (2 sites) in 2002–2003 (Table 1), stored whole at −20°C, and then individually processed for DNA extraction using the QIAamp Mini Kit (Qiagen, Hilden, Germany) as described in Mixson et al. [14]. These tick samples were not pre-treated prior to DNA extraction to reduce surface contaminants as they were collected and processed under older protocols for a different purpose than this experiment. DNA extraction was performed during 2002–2004, and the DNAs were stored at 4°C until used in this study. DNAs extracted following this manufacturer’s protocols are stable at least 16 years at 4°C [29].

**Table 1:** Life stage and geographic origin of *Amblyomma americanum* ticks used in bacterial community analyses.

Tick DNA Set	Collection Site (Year)	Nymph	Adult Male	Adult Female	Total
Archival	Bodie Island, NC (July 2002)	NT	5	5	10
Archival	Buxton Woods, NC (July 2002)	NT	4	5	9
Archival	Shelter Island, NY (July 2003)	NT	3	5	8
Archival Total		NT	12	15	27
Georgia	Panola Mountain, GA (2010–11)§	30	8	14	52
Georgia	Sweetwater Creek, GA (2010–11)*	36	4	12	52
Georgia Total		66	12	26	104

§ Nymphs collected in July 2010; adults in July 2010 and May 2011.

*Nymphs collected in August 2010; adults in May 2011.

NT = Not tested.

### 454 library preparation and sequencing

PCR primer and barcode designs were obtained from the Human Microbiome Project’s 16S 454 Sequencing Protocol. The primer sequences included eubacterial *rrs* primers 357F and 926R, which target variable regions 5–3 [30]. Five prime modifications of primers were made for compatibility with the Roche (Indianapolis, Indiana, USA) 454 sequencer’s Titanium chemistry. Modified primer sequences were F: 5′-CTA TGC GCC TTG CCA GCC CGC TCA GCC TAC GGG AGG CAG CAG-3′ and R: 5′-CGT ATC GCC TCC CTC GCG CCA TCA G (barcode) CCC GTC AAT TCM TTT RAG T-3′. Barcode sequences are provided in the supporting information (Table S1). The expected amplicon size was approximately 643 bp.

For PCR, each 20 µL reaction mix contained either 1x AccuPrime PCR Buffer II (Invitrogen, Carlsbad, California, USA), 0.5 U AccuPrime Taq High Fidelity, 13.5 µL water, 0.3 µM each forward and reverse primer, and 2 µL DNA or 15 µL Platinum PCR SuperMix High Fidelity (Invitrogen), and 1 µL water. We have found no significant differences in bacterial community composition when using these two enzymes for primary PCR (data comparing tick DNAs amplified with both polymerases are described further below and in the results). All reactions were performed in an Eppendorf Master Gradient thermocycler (Brinkmann Instruments, Westbury, New York, USA) with the following program: one step of 94°C (2 min), 35 cycles of 94°C (30 s), 50°C (30 s), and 68°C (1 min), and one step of 68°C (5 min), followed by holding at 4°C. Amplicons were analyzed by electrophoresis on 1% agarose gels stained with ethidium bromide to ensure reaction success and then quantitated using the Quant-iT PicoGreen dsDNA kit (Invitrogen) modified from the manufacturer’s protocol to 50% of the recommended final assay volume. Equal quantities of each PCR product were pooled, and the pool was purified using the Wizard SV Gel and PCR Clean-Up System (Promega). The pool was sequenced by the Centers for Disease Control and Prevention (CDC) Biotechnology Core Facility Branch on a Roche 454 GS-FLX sequencer.

To determine if the two polymerases that we used differentially biased the bacterial communities identified from these tick DNAs, we amplified 50 tick DNAs belonging to the archival collection once with each of the polymerases used in this experiment as described above. Tick DNA and barcode pairings were held constant between the libraries. A single library was produced from the AccuPrime amplicons, and two libraries were produced from the Platinum amplicons to control for variability introduced by random sampling, pipetting error, and bead loading on the 454 sequencer. Each library was sequenced on one quarter of a plate (Table S2). Twenty-six of 50 DNAs were sequenced to a depth of at least 1000 high quality reads across all three treatments and were used in the analysis of polymerase effects.

Life stage effects were analyzed using amplicons from six quarters from three sequencing plates (Table S2). Some DNAs were sequenced more than once, but no duplicates were used in the analyses. Samples not analyzed here were replicates of reported samples, belonged to variable groups that were not sufficiently sampled, or returned insufficient numbers of high quality sequences for further analyses.

### Bioinformatic analysis

The bioinformatic pipeline was executed in the software package mothur (version 1.31.2) [31]. The quality control pipeline was modeled on the Schloss SOP pipeline available on the mothur website [32]. Briefly, flow data were denoised and the resulting sequences were discarded if the reverse primer sequence or barcode could not be identified, or if barcode or primer sequences had more than 1 or 2 errors, respectively. Sequences were also discarded that contained homopolymer runs >8 bp or ambiguous nucleotide calls. Sequence trimming was performed based on a sliding window of 50 bp with a threshold quality score of 35. The remaining sequences were aligned to the SILVA SEED bacterial 16S database [33] using 8mer searching to choose a template sequence and the Needleman-Wunsch algorithm to perform the alignment. The SILVA SEED database was modified to include several sequences of interest, eg. *Arsenophonus* and *Candidatus* ‘Midichloria mitochondrii’. Aligned sequences were then screened by starting positions to eliminate sequences that mapped outside the target region of the *rrs*. Finally, chimeric sequences were identified using the chimera.uchime function in mothur and then removed from further analyses.

All sequences passing the quality control pipeline were assigned to operational taxonomic units (OTUs) as described in the Schloss SOP. To summarize, all high quality sequence reads were ranked by abundance and rare reads differing from abundant reads by a single base were assumed to be sequencing errors and clustered with their similar sequence. An uncorrected pairwise distance matrix was calculated between the aligned sequence reads; both internal and terminal gaps were penalized once. Reads were then clustered into OTUs using the average neighbor method at a 3% distance. Taxonomic assignment of individual reads was determined by using mothur’s Bayesian classifier and requiring a bootstrap confidence of 80% on 100 iterations (kmer size = 8). A consensus taxonomy was determined for each OTU based on the individual sequence taxonomies within each. Genus level identification is provided whenever possible; otherwise the lowest taxonomic rank applying to all members of the OTU is given. Singleton OTUs were removed prior to statistical analyses [34]. Representative highest abundance sequences from OTUs are deposited in GenBank under accession numbers KJ130495–KJ130517; additional information can be found in Table S3. The full data set is available in the NCBI SRA under study accession number SRP042723.

### Bacterial diversity analyses

Good’s community coverage estimate [35], rarefaction curves, and alpha diversity were calculated in the software mothur using a subsample size of 1000 sequences and 1000 iterations. The inverse Simpson diversity index (1/D) was used for alpha diversity because it has a clear biological interpretation and is less affected by sample size [36] and the presence of spurious OTUs [37] than similar measures. Analysis of variance (ANOVA) and Tukey’s honestly significant difference (HSD) post hoc tests were performed on the R statistical platform (version 3.0.1) [38].

### Bacterial community analyses

We used community ecology methods to analyze both the OTU composition (presence/absence) and structure (abundance) of bacterial communities observed within each tick. As no one metric adequately compares all aspects of community assemblages, we measured the distance between bacterial communities using two metrics that each emphasize different community characteristics. The Jaccard dissimilarity index performs pairwise comparisons of communities based on the presence or absence of OTUs [39], while the Bray-Curtis dissimilarity index weights the abundance of OTUs in its calculations [40]. Each tick bacterial community was rarefied to 1000 sequences, and Jaccard and Bray-Curtis calculations were performed in R with the package “vegan” (version 2.0–7) [41] using the vegdist command.

To partition variation within each distance matrix, we used a non-parametric permutational multivariate analysis of variance (PERMANOVA) as implemented in the vegan function adonis (permutations = 1000) [41]. As PERMANOVA can sometimes be affected by differences in within group variation, each matrix was also tested for homogeneity of group dispersions via the vegan function betadisper [41]. Significance values were obtained by permutation (n = 1000). Corrections for multiple comparisons in the PERMANOVA and dispersion tests were performed using Holm’s method [42].

To visualize data sets and to corroborate the results of the PERMANOVAs, a non-metric multi-dimensional scaling (NMDS) was performed on each distance matrix. This ordination technique represents highly dimensional data by maximizing the correlation in rank-order between the original data set and a two dimensional representation [43], [44]. The relative location of each tick’s bacterial community within the ordination space can be interpreted with the addition of species (treating OTUs as species for this purpose) scores to the plot. Bacterial communities closer to a given species score in ordination space have greater values for that species than those communities located farther away [45]. The ordinations were produced using the function metaMDS in vegan (maximum permutations = 1000) with square root and Wisconsin double standardization transformations used according to default settings [41]. The goodness of fit for each variable’s group centroids were evaluated using the command envfit [41], and the species scores were calculated as weighted averages of the bacterial community scores [41]. Groups are considered significantly different for α = 0.05 if the 95% confidence intervals did not overlap.

To determine which OTUs differed in abundance between groups we used the Metastats method of White et al. [46] as implemented in mothur. In an effort to control type II error rates, we only interpreted those *p*-values that had an associated false discovery rate (i.e., the proportion of false positives expected in a set of reported significant results) of *q*≤0.05 [46], [47].

### Polymerase effects analysis

For the archival tick DNAs successfully sequenced across the AccuPrime, Platinum 1, and Platinum 2 libraries, we processed the data as described above in bioinformatic analysis, rarefied each community to 1000 sequences, and compared the bacterial communities from each library originating from the same tick DNA. Jaccard and Bray-Curtis dissimilarity values were calculated pairwise for each tick’s three bacterial communities. For each dissimilarity metric an ANOVA was used to compare the means of each of the three comparison groups (Platinum 1 and Platinum 2, Platinum 1 and Accuprime, Platinum 2 and Accuprime) across ticks. The means would differ only if a polymerase produced a differentially biased bacterial community composition or structure as measured by the Jaccard or Bray-Curtis metrics, respectively. Bartlett’s test [48] was used to ensure homogeneity of group variances. All statistics were performed on the R platform.

### Co-infection analysis

The co-occurrence of the most abundant OTUs (≥100 sequences in the rarefied data) was evaluated using a probabilistic model to detect pairs that occurred together significantly more or less often than if OTU assemblages were random [49]. The analysis software was kindly provided by the test’s author (v1.0). The Pearson correlation coefficient (*r*) was calculated using the cor.test function in the R statistical platform [38] with a two-sided alternative hypothesis.

## Results

### Effect of Polymerase on Bacterial Community Diversity

The Accuprime polymerase library produced 114,250 raw sequences compared to the 82,843 and 93,834 sequences produced by the Platinum polymerase libraries. The distribution of raw sequence lengths (Figure S1), the proportion of sequences removed as sequencing errors, alignment errors, chimeras, and contaminants (Figure S2), and the proportion of sequences retained as high quality sequences (Figure S2) were similar between the polymerases. Bartlett’s test for homogeneity of variances was non-significant for both the Jaccard and Bray-Curtis metrics. ANOVA detected no difference in the mean level of dissimilarity between bacterial communities originating from the same tick DNA but amplified with different polymerases (Platinum 1 and Accuprime, Platinum 2 and Accuprime) and bacterial communities that originated from the same amplification of the same tick DNA (Platinum 1 and Platinum 2) (Jaccard: *F*(2,75) = 2.74, *p*>0.07; Bray-Curtis: *F*(2,75) = 1.40, *p*>0.25). Because the AccuPrime and Platinum SuperMix polymerases did not differentially bias bacterial community detection, bacterial communities amplified using these polymerases were pooled in all life stage analyses below.

### Life stage library characteristics

We obtained 350,501 high quality 16S rRNA gene sequence reads from 131 *A. americanum* tick samples from five sites (Tables 1 and S2). These sequences belonged to two tick DNA sample sets, referred to as the archival and Georgia tick DNA sets. Additional tick DNA samples sequenced on the same plates were not included in the analyses because they were inferior replicates of retained tick samples, produced low sequence yields (<1000), or belonged to variable groups that were insufficiently sampled for analysis. The data were dominated by sequences from high-abundance OTUs belonging to *Rickettsia* and *Coxiella*, which comprised 35.0% and 59.4% of sequences, respectively, and were found in 88.5% and 99.2% of the ticks, respectively. The sequence reads averaged 255.6 bp in length after trimming and comprised 408 operational taxonomic units (OTUs) when clustered at 97% identity. One hundred seventy OTUs were comprised of a single sequence (singletons) and were removed [34]. To facilitate comparison of bacterial communities between samples, each bacterial community was then rarefied to 1000 sequences. This reduced the total number of OTUs to 212, of which 42 contained only a single sequence. The 170 OTUs containing >1 sequence represented a minimum of 99 bacterial families and 99 genera.

### Bacterial diversity

The Georgia DNA set’s rarefaction curve approached an asymptote, indicating that the available bacterial diversity was well sampled (Figure S3). This conclusion was supported by the Good’s coverage estimate for each tick sample, which had a mean of 0.998 (range 0.994–1.00). In contrast, the bacterial diversity of the archival DNAs, from ticks which were not surface decontaminated prior to DNA extraction, was less well sampled and the rarefaction curve had a steeper slope. However, while adding additional tick DNAs samples to this set would have detected additional OTUs, the communities of each tick in the data set were individually well sampled (Good’s coverage estimate, mean = 0.993, range = 0.976–1.00).

**Figure 1:**
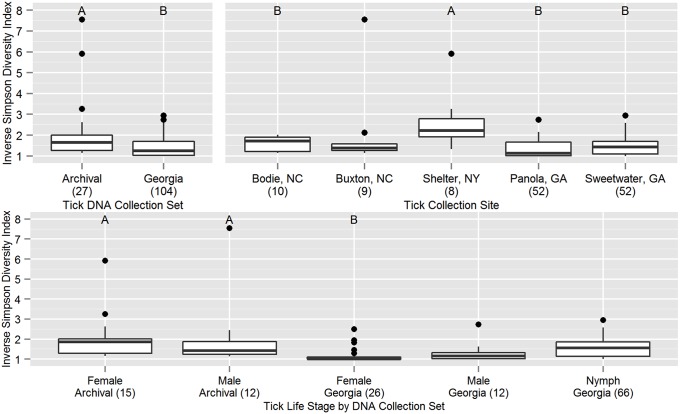
Comparisons of bacterial community alpha diversity by tick DNA set, life stage, and collection site. Box and whiskers plots represent the first and third quartiles (hinges), the median (bold line), and 1.5 times the interquartile range (error bars). Outlier points are plotted individually. Within each of three panels, n = 131 (see Table 1) and groups labeled A are significantly different from groups labeled B at α = 0.05 (Tukey’s HSD). Group sample sizes are given in parentheses after the group name. For the tick DNA sample set comparison, the archival tick DNAs were older and processed under different extraction protocols than the Georgia samples.

Alpha diversity was estimated using the inverse Simpson index and compared between DNA sample sets and across life stages and collection sites (Figure 1). Significant differences were found for all variables considered (ANOVA with Holm’s correction for multiple comparisons, DNA collections: *F*(1,129) = 15.9, *p* = 0.00023; life stages: *F*(4,126) = 5.21, *p* = 0.00064; sites: *F*(4,126) = 6.83, *p* = 0.00016). Among life stages from the different DNA collection sets, adult females from Georgia had lower alpha diversity than adult males (Tukey’s HSD, *p* = 0.013) and females (*p* = 0.0017) from the archival DNA collection. Comparisons across collection sites found a higher alpha diversity among ticks from Shelter Island compared to those from Bodie Island (Tukey’s HSD, *p* = 0.022), Sweetwater Creek (*p* = 0.00059) and Panola Mountain (*p* = 6.9×10^−5^).

### Bacterial community composition and structure

**Figure 2:**
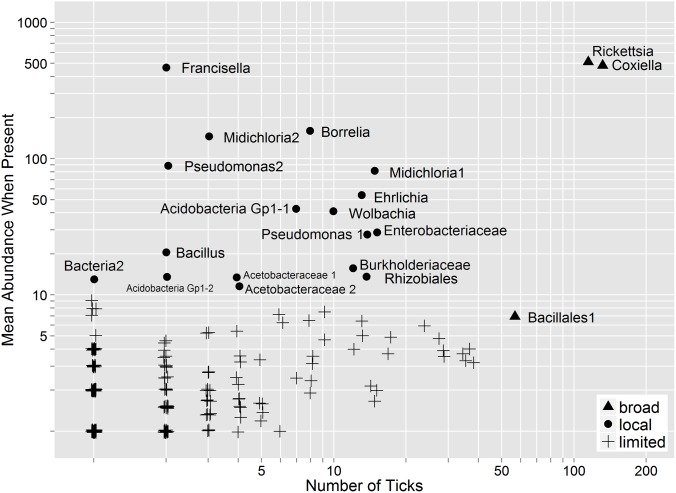
Bacterial operational taxonomic unit (OTU) sequence abundance detected in tick samples. OTUs present in greater than one third of all tick samples were considered broadly distributed. OTUs present at a mean abundance ≥1% of the community (i.e. 10 sequences) were considered locally abundant. Sequence abundance values are slightly offset to reveal overlapping OTUs.

Of the 212 OTUs analyzed across both DNA collections, only *Rickettsia*, *Coxiella*, and a Bacillales group were observed in more than one third of all standardized samples (Figure 2). An additional 17 OTUs composed ≥1% of the community when detected, but were detected in one third or fewer of the tick DNA samples. No individual OTU was present in all rarefied samples, including *Coxiella*, a proposed obligate symbiont of *A. americanum* [21]. Of the two negative samples, the male from Panola Mountain was *Coxiella* positive prior to rarefaction, while the nymph from Sweetwater Creek was positive in a replicate sequencing run (data not shown).

DNAs from ticks collected in Georgia differed from those in the archival DNA collection in the age of the samples, the tick life stages represented, and the DNA extraction protocols used. Bacterial community structure was quite different between these two groups of ticks (PERMANOVA on Bray-Curtis distance matrix, *F* = 5.87, R^2^ = 0.044, *p* = 0.012) (Figure S4), but they did not differ in their dispersion (*F* = 1.09, *p* = 0.28). The bacterial communities of the Georgia tick DNAs contained 12 OTUs not found in the archival tick DNA bacterial communities, while the archival tick DNAs contained 61 OTUS not found among the Georgia tick bacterial communities. An additional 7 OTUs were present in both DNA collections but differentially abundant (Table S4). Greater than 93% (75/80) of the OTUs that were differentially abundant between the Georgia and archival tick DNAs have no confirmed association with the internal microbiota of ticks and therefore may have been derived from the external environment.

**Figure 3:**
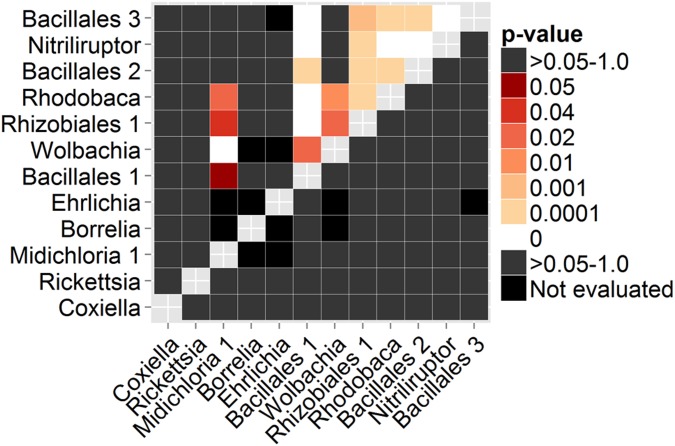
Significance of co-infection patterns between selected operational taxonomic units (OTUs). Only data from the Georgia tick DNA sample collection is shown. The upper triangle represents *p*-values of positive associations (codetection in the same sample) between OTUs and the lower triangle the *p*-values of negative associations. Black cells were not evaluated due to low expected co-occurrence (<1) between the OTUs [49].

**Figure 4:**
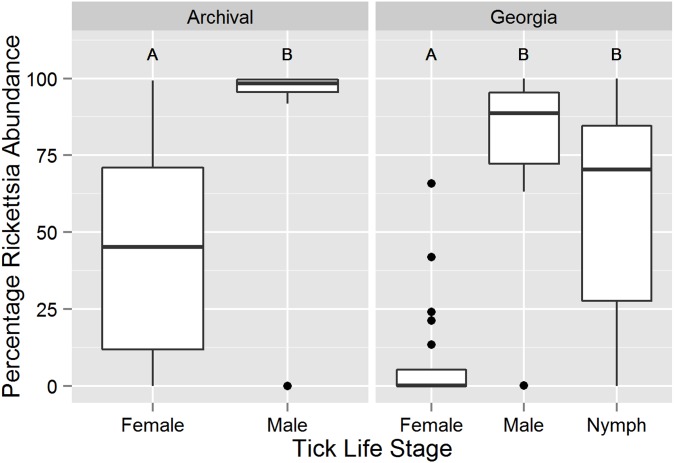
Variation in percentage of *Rickettsia* among total *Rickettsia* and *Coxiella* sequences by tick life stage. The number of unstandardized *Rickettsia* and *Coxiella* sequences from each tick sample (Georgia n = 104, archival n = 27) were summed and the percentage of *Rickettsia* sequences were calculated. Box and whiskers plots represent the first and third quartiles (hinges), the median (bold line), and 1.5 times the interquartile range (error bars). Outlier points are plotted individually. Within each DNA collection, life stages labeled with different letters are significantly different at α = 0.05.

Twenty-two positive associations were detected among the 12 most abundant (>100 sequences) OTUs from the Georgia DNA collection (Figure 3). The Bacillales 1, *Rhodobaca*, and Rhizobiales 1 OTUs had the largest number of associations. The *Wolbachia* and *Midichloria* 1 OTUs were strongly positively associated. Two positive associations were found among the most abundant OTUs from the archival DNA collection (Rhizobiales and Acidobacteria Gp1, Burkholderiaceae and *Methylobacterium*, *p*<0.04). In general no significant associations were detected between the *Rickettsia* and *Coxiella* OTUs and the other OTUs due to the near ubiquity of *Rickettsia* and *Coxiella* in these samples. However, there was a significant inverse relationship between the abundance of *Rickettsia* and *Coxiella* across both sample sets (Georgia: n = 104, Pearson’s *r* = −0.976, *p* = 2.2×10^−16^; archival: n = 27, Pearson’s *r* = −0.689, *p* = 6.97×10^−6^). The life stages differed in the dominant bacteria (ANOVA on percentage *Rickettsia* out of total *Rickettsia* and *Coxiella* sequences, Georgia: *F*(2,101) = 26.8, *p* = 4.68×10^−10^; archival: *F*(1,25) = 14.0, *p* = 0.00097) (Figure 4). Within the Georgia collection, females differed from males and nymphs (Tukey’s HSD, males: *p* = 1.0×10^−7^, nymphs: *p*<1.0×10^−7^) (Figure 4).

### Life stage and sex specific bacterial communities

**Figure 5:**
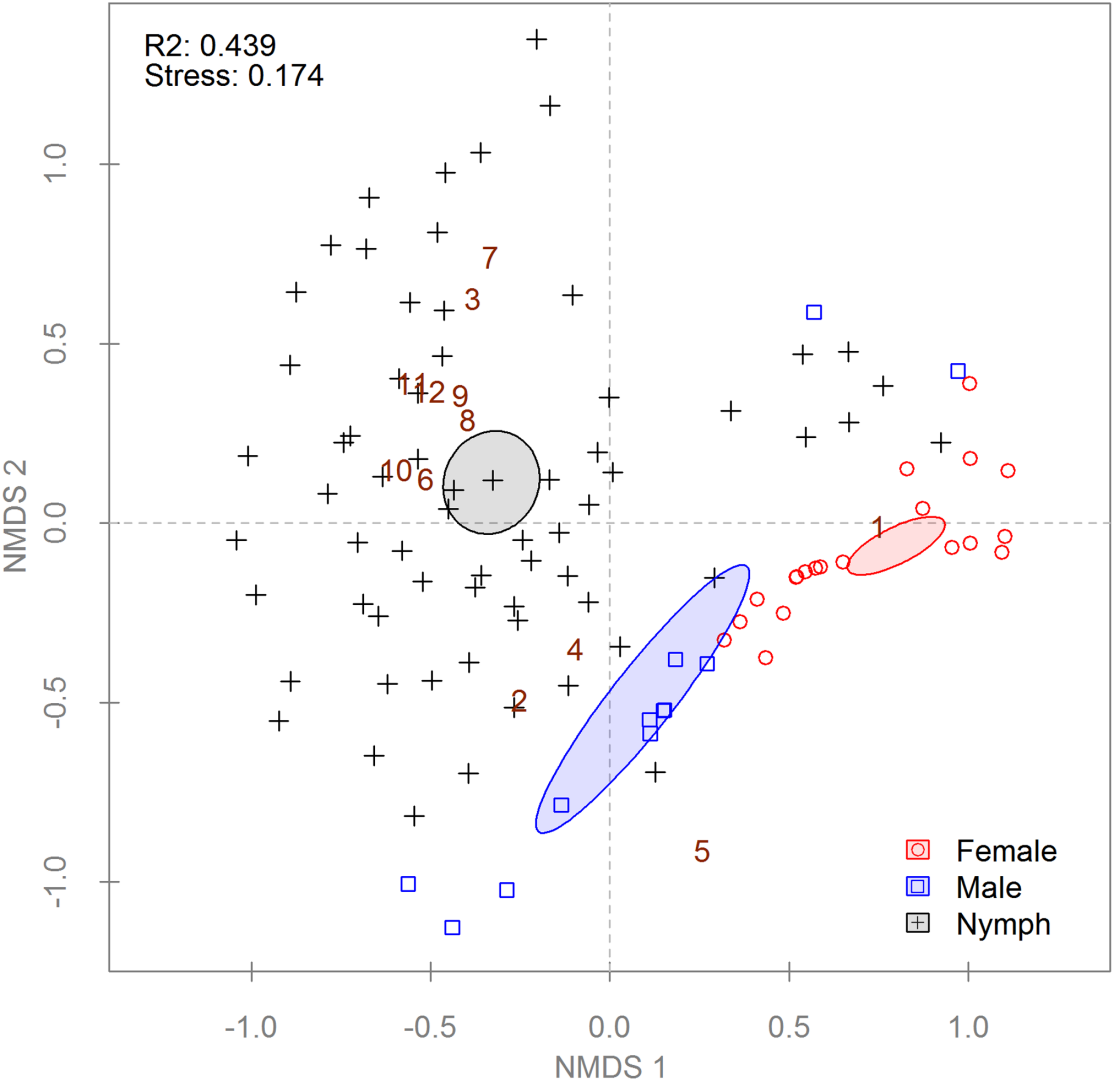
Non-metric multi-dimensional scaling (NMDS) of a Bray-Curtis distance matrix describing Georgian *Amblyomma americanum* bacterial communities. Each point symbolizes a single tick’s community (n = 104); some points may overlap completely. Point and ellipse colors indicate life stage; ellipses represent 95% confidence intervals around life stage centroids. Non-overlapping centroids are considered significantly different at α = 0.05. R^2^ values in the upper left corner of plots describe the amount of variation in the data set explained by the groupings. The stress value given is a measure of the disagreement between the rank order in the original data set and that in the NMDS (lower numbers indicate better agreement). Brown numbers indicate the species scores for select OTUs as follows: (1) *Coxiella*, (2) *Rickettsia*, (3) *Midichloria* 1, (4) *Borrelia*, (5) *Ehrlichia*, (6) Bacillales 1, (7) *Wolbachia*, (8) Rhizobiales 1, (9) *Rhodobaca*, (10) Bacillales 2, (11) *Nitriliruptor*, (12) Bacillales 3.

Ticks from the Georgia collection were analyzed to detect differences between female, male, and nymphal bacterial communities (n = 104). Samples from Sweetwater Creek and Panola Mountain, Georgia were pooled because no differences were detected between the sites (data not shown). PERMANOVA found significant differences between life stages and sexes using both distance metrics (Table 2). Differences in within group variation (i.e. dispersion) were also detected with both distance matrices (Table 2) [50]. Results from the PERMANOVA were corroborated using ordination by NMDS. When only the presence or absence of OTUs was considered (Figure S5), nymphal communities were different from adults, but male and female communities did not differ. However, differences were detected between males and females when the ordination was weighted for OTU abundance (Figure 5). Comparison of the mean relative abundance of OTUs detected 8 of 13 which were differentially abundant between the tick life stages and sexes (Figure S6).

**Table 2:** Multivariate analysis of the effect of sex and life stage on the relatedness of *Amblyomma americanum* bacterial communities.

			PERMANOVA			Dispersion Test	
Collection	Distance Metric	Metric Type	*F*	R^2^	*p* *	*F*	*p* *
Georgia	Jaccard	Community composition	17.3	0.255	0.0020	12.5	0.0020
Georgia	Bray-Curtis	Community structure	24.9	0.330	0.0020	8.77	0.0020
Archival	Jaccard	Community composition	1.63	0.0611	0.026	0.533	0.96
Archival	Bray-Curtis	Community structure	8.28	0.249	0.0020	0.345	0.96

Tick DNAs from the archival collection (n = 27) were older, collected in a different geographical region, and processed using different protocols than tick DNAs from the Georgia collection (n = 104).

*Corrected for multiple comparisons using Holm’s method; α  = 0.05.

**Figure 6:**
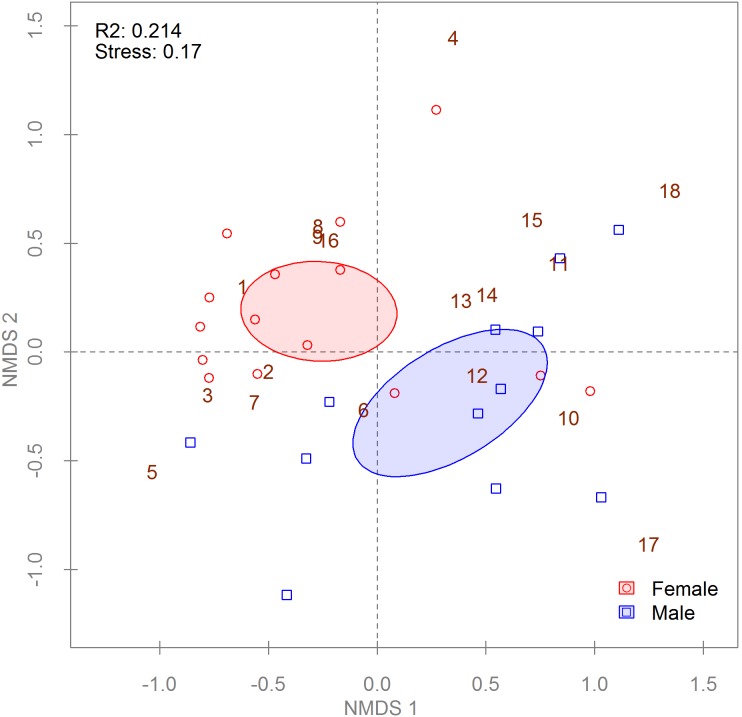
Non-metric multi-dimensional scaling (NMDS) of a Bray-Curtis distance matrix describing archival *Amblyomma americanum* bacterial communities. Each point symbolizes a single tick’s community (n = 27); some points may overlap completely. Point and ellipse colors indicate life stage; ellipses represent 95% confidence intervals around life stage centroids. Non-overlapping centroids are considered significantly different at α = 0.05. R^2^ values in the upper left corner of plots describe the amount of variation in the data set explained by the groupings. The stress value is a measure of the disagreement between the rank order in the original data set and that in the NMDS (lower numbers indicate better agreement). Numbers indicate the species scores for the plot as follows: (1) *Coxiella*, (2) *Rickettsia*, (3) *Midichloria* 1, (4) *Borrelia*, (5) *Francisella*, (6) *Midichloria* 2, (7) *Ehrlichia*, (8) Enterobacteriaceae, (9) *Pseudomonas* 1, (10) Burkholderiaceae, (11) Acidobacteria Gp1, (12) *Pseudomonas* 2, (13) Rhizobiales 2, (14) *Bacillus*, (15) Acetobacteraceae 1, (16) Acetobacteraceae 2, (17) Bacteria 1, (18) Bacteria 2.

To determine if an effect of sex on tick bacterial communities could be detected in other parts of the *A. americanum* range, we applied PERMANOVA to Jaccard and Bray-Curtis distance matrices with data from the archival DNA samples from adult ticks collected outside of Georgia (n = 27) (Table 1). Ticks from all three sites were pooled for analysis because no site effect was detected (data not shown). PERMANOVA found significant differences for both distance matrices (Table 2), but NMDS found there was a difference between males and females only in the relative abundance of bacterial community members (Bray-Curtis metric, Figure 6) and not in community membership (Jaccard metric, Figure S7). Analysis of the OTUs containing ≥100 sequences each found 2 of 18 were differentially abundant between groups, with females possessing significantly higher *Coxiella* abundance and having higher *Francisella* abundance (*p*<0.001).

## Discussion

454 pyrosequencing of barcoded *rrs* variable region 5–3 amplicons was used here to characterize the bacterial communities of 131 individual *A. americanum* ticks, including nymphs and adult males and females. The ticks were all questing, field-collected individuals obtained from five sites in three states; as such they are probably at least representative of the diversity of bacterial communities that occur in *A. americanum* found in the eastern states comprising its range.

The exact relationship of 454 sequence read abundance to the biological abundance of a given bacteria in the community is difficult to prove as amplification efficiency varies both between bacterial targets (e.g. template GC content [37], primer-template matching [51], 16S copy number variation per chromosome and per cell) and laboratory protocols (e.g. primer bar-code bias [52], PCR conditions [53]). In this system, while previous work has shown a good correlation between 454 read abundance and targeted qPCR quantitation [54], it is not currently possible to confirm such a relationship for all potential members of the *A. americanum* bacterial community. In general, while comparing communities across protocols is problematic due to the many potential sources of bias, 454 metagenome results are reproducible for a given protocol [37], [55], [56]; however, some authors have found that this is not always true [34].

We document here significant differences in the bacterial communities of the life stages and sexes of questing *A. americanum*. Males and females in both the Georgia and archival DNA collections differed in their bacterial community structure (Figures 5 and 6) but not composition (Figures S5 and S7). However, nymphs differed from adults in both bacterial community composition and structure (Figures S5 and 5, respectively), with many of the more abundant OTUs appearing primarily in nymphs (Figure S6). Many, but not all, of these OTUs belonged to taxa with both environmental and arthropod-associated members. While the nymphs from Georgia were surface decontaminated prior to DNA extraction, our method was unlikely to remove all exoskeleton-associated bacteria. The higher surface to volume ratio of nymphs compared to adults also provides a larger relative area for contamination. However, Menchaca et al. [22] found high levels of Bradyrhizobiaceae in both nymphs and adults derived from laboratory colonies and held indoors, suggesting a more persistent association for at least one of these taxa.

As we expected from previous investigations of the bacterial community of *A. americanum* [14], [16], [19], [22], [57], [58] (Table S3), *Rickettsia* and *Coxiella* were by far the dominant OTUs identified in our samples across all life stages, sexes, and DNA collections. The *A. americanum*-associated *Coxiella* is regarded as an obligate symbiont of the lone star tick due to its ubiquitous presence in previous surveys, limited evidence for a reduced genome size relative to the free-living relative (*Coxiella burnetii*), transovarial and transstadial maintenance, and the reduced viability and fecundity of antibiotic treated ticks [16], [20], [21], [59]. While one tick had no detectable *Coxiella* sequences in the data presented here, we later detected low levels of *Coxiella* in a replicate data set. This suggests that the abundance of *Coxiella* was at the limit of detection for 454 in this sample. Our data provide further support for the hypothesis that *Coxiella* is an obligate symbiont of *A. americanum*, although its population size in some individuals may be very low relative to other members of the bacterial community.

Fragments of the *rrs* are insensitive for the identification of different species of *Rickettsia*. However, abundant previous work has shown that the most common *Rickettsia* in *A. americanum* is *R. amblyommii* (eg. [14], [16], [57], [58], [60], [61]), a member of the spotted fever group with poorly defined human pathogenicity [62]–[64]. Despite efficient vertical [65] and probable horizontal (reviewed in [66]) transmission in *A. americanum*, infection rates in populations across the tick’s range vary widely (0–84%) [14], [16], [60], [61]. Our archival tick DNAs (Table 1) had been previously tested for *R. amblyommii* using direct PCR of the gene encoding the rOmpA protein followed by restriction fragment length polymorphism analysis; techniques and data were described in Mixson et al. [14]. Of these 27 adult ticks, two were negative by direct PCR but contained at least a few *Rickettsia* sequences when analyzed here (n = 1–160 sequences per tick, original unrarefied data). While *R. amblyommii* generally occurs as a high density, disseminated infection in *A. americanum* [67], taken together these data indicate that some *R. amblyommii* infections are below the detection limit of direct PCR and may not always be reliably detected by that insensitive technique (this has been affirmed by qPCR comparison of varying and often low *R. amblyommii* levels in other tick samples, Dasch unpublished results). *Rickettsia amblyommii* infection rates are therefore likely to be somewhat higher than has been previously reported, although whether or not the bacterium is actually ubiquitous or transcriptionally active in all samples remains to be determined. If naturally uninfected ticks exist, these low level infections may represent individuals that had acquired the bacterium during the preceding blood meal.

There was a strong inverse relationship between the abundance of *Rickettsia* and *Coxiella* in the bacterial communities of the adult *A. americanum* ticks from both the Georgia and archival DNA collections. Interestingly, the dominant bacterium varied with the sex of adults such that females produced more *Coxiella* sequences and males more *Rickettsia* sequences (Figure 4). The relatively higher *Coxiella* to *Rickettsia* ratio observed in females is supported by the qPCR data of Jasinskas et al. [20], but differs from the report of Ponnusamy et al. [18], who detected an increase in *Rickettsia* in females and low levels of *Coxiella* across all *A. americanum* samples using pyrosequenced V1-3 *rrs* fragments. Given the extensive evidence for *Coxiella*’s abundance in most *A. americanum* samples [16], [17], [19], [22] and the small sample analyzed by Ponnusamy et al., it is likely that either some bias against *Coxiella* existed in the Ponnusamy et al. protocol or that a non-representative sample was drawn from their population. If *Coxiella* is obligate for the survival of *A. americanum* [21], the number of bacteria may increase in females to ensure 100% transmission to eggs. An alternative, but not mutually exclusive, explanation is that males may contain higher numbers of *R. amblyommii* to facilitate increased locomotion [68], which would increase reproductive fitness if it enabled more mating opportunities for males. The distribution of nymphal bacterial communities across the gradient from *Rickettsia* dominated to *Coxiella* dominated suggests that sex-biased bacterial communities may arise in unfed questing nymphs (Figure 4). It will be difficult to prove this hypothesis until molecular sexing tools are available for *A. americanum*.

The only previously documented difference in *A. americanum* bacterial communities was decreased species richness in females relative to males and nymphs [18], but similar patterns to those reported here have been noted for other ixodid tick species. In *Rhipicephalus turanicus*, *Coxiella* sequences composed 90% of the total detected by bacterial 16S pyrosequencing in both males and females, but *Rickettsia* were relatively more abundant in infected males than in females [25]. Males also had greater bacterial richness than females, although there was no difference in diversity. In *Ixodes scapularis*, Moreno et al. used temporal temperature gradient gel electrophoresis of bacterial 16S amplicons to “finger print” the bacterial communities of ticks and found differences between nymphs, males, and females [23]. *Francisella* have been shown to increase in *Dermacentor variabilis* nymphs relative to larvae using 16S amplicon pyrosequencing [24]. Studies of individual bacteria have also revealed sex and life stage biased prevalence. For example, *Candidatus* ‘Midichloria mitochondrii’ (hereafter “*M. mitochondrii*”) is detected in only 40% of males but 100% of all other life stages of *Ixodes ricinus* [27]. *Borrelia* and *Anaplasma* infection rates also differ between male and female *I. ricinus* [26].

We also identified other potentially important intracellular bacteria in *A. americanum*. Two OTUs of *M. mitochondrii* were found, which contained all three of the genotypes detected from *A. americanum* in our previous work [8]. Operational taxonomic unit *Midichloria* 1 corresponds to genotypes A and B, and OTU *Midichloria* 2 is equivalent to genotype C. *Wolbachia* sequences were also identified in *A. americanum* from Georgia. *Wolbachia* have been previously reported in *A. americanum* by Zhang et al. [69], but a direct comparison is not possible because the two studies used different regions of the *rrs* gene. *Wolbachia* sequences were detected in our unrarefied data from 11 nymphs and 2 females collected from the two Georgia sites (Panola Mountain nymphs: 4/30, females: 2/14; Sweetwater Creek nymphs: 7/36, females: 0/12). These infection rates were at least two times greater than the highest minimum infection rates previously observed in nymph and female populations in Maryland [69], which could be a result of the increased sensitivity of Next-Gen sequencing versus direct PCR, the dilution effect of pooling of nymphs in earlier work, or simply a statistical artifact of our smaller sample sizes. As in previous work [69], we detected no *Wolbachia-*positive males, although our sample sizes for males were lower than those for females and nymphs. These *Wolbachia* have been previously typed as supergroup F [69], which also contains *Wolbachia* that infect scorpions [70], lice [71], filarial nematodes [72], and other hosts (reviewed in [73]). Interestingly, 11/13 of these *Wolbachia* positive ticks were also positive for genotype A of *M. mitochondrii*, which to date has only been found in Georgia [8]. This co-infection was highly significant, despite the co-clustering of *M. mitochondrii* genotype A in OTU *Midichloria* 1 with genotype B (*p*<0.0001) (Figure 3). There are at least three potential mechanisms that may lead to this degree of co-infection, including (i) stable vertical transmission of both bacteria within one or more tick lineages, (ii) repeated, simultaneous horizontal transmission of both bacteria from a single source, or (iii) differential survival of *M. mitochondrii*-infected ticks when attacked by *Wolbachia*-bearing insects or nematodes. Further work will be needed to differentiate between these hypotheses.

The bacterial OTUs that we detected across all tick samples were very similar to those identified by Yuan [19], who also used wild caught ticks and similar sequencing methods. Our results also did not differ substantively from those of Clay et al. [16] or Heise et al. [17], except for the absence of *Arsenophonus* in our samples [16]. However, our results were generally quite different from the previous work of Menchaca et al. [22], despite having used a similar fragment of the *rrs*. While both studies detected *Coxiella*, the ticks from the previous study contained a large proportion of taxa found rarely or not at all in our data, such as Clostridia, Caulobacteraceae, and Hyphomicrobiaceae. Whether these differences result from the different methods used by Menchaca et al. (Ion Torrent sequencing of nested PCR products), the use of laboratory raised ticks, or their exclusive feeding of ticks on a domestic chicken remains to be determined. However, some evidence for *D. variabilis* and *I. scapularis* ticks suggests that the vertebrate hosts have little effect on bacterial community structure [24], while some laboratory colonies of ticks have been observed to differ in some members of their bacterial community [17].

Most of the bacterial taxa identified in this study are broadly distributed in the environment with members found in soil, plants, and arthropods (Table S3). Many were previously identified in similar deep sequencing studies of other tick species (Table S3), but the low taxonomic resolution provided by short *rrs* fragments for these very diverse groups does not preclude similar surface contaminants on ticks collected on different continents. The enrichment of bacterial communities from the archival tick DNAs extracted without surface disinfection with potential environmental bacteria (Table S4) suggests that they are indeed contaminants, but the confounding variables of geographic location, DNA extraction methods, and tick life stage prevent definitive analysis here. Previous comparisons of surface decontaminated versus untreated *A. americanum* adults supports Sphingobacteria as a surface contaminant, but not other high abundance OTUs [22]. Several of these OTUs (Actinobacteria, *Pseudomonas*, Bacillales, Rhizobiales, and Burkholderiaceae) were also commonly found in *A. americanum* samples collected in the Midwestern USA [16], Texas and Missouri [19], and laboratory colonies [17]. Further work with bacterial species-specific methods should be undertaken to determine the topological relationship of these bacteria to ticks.

In summary, we have found a pattern of sex-specific bacterial community structure that was present irrespective of geographic origin of our *A. americanum* samples and significant differences in both the composition and structure of nymphal bacterial communities relative to adults. We also documented a novel association between *M. mitochondrii* and *Wolbachia* in ticks from Georgia. Given the innate limitations of the sequencing of *rrs* amplicon fragments, this metagenomic work has been effective as a discovery tool for future targeted studies. However, the extreme dominance of *R. amblyommii* and *Coxiella* in the *A. americanum* bacterial community makes it difficult to detect and analyze other community members in a statistically robust way. Future metagenomic studies would benefit from targeted depletion of the most prevalent and predominant bacteria to increase detection rates for other rarer but potentially important community members.

## Supporting Information

Figure S1 **Comparison of raw 454 read length distributions between replicate libraries created with different polymerases.** Each library was produced with the same set of DNA templates and barcoded primers. The polymerase varied between panels A and B/C, while B and C were produced from the same amplification products but pooled and sequenced independently. Panel A represents the library created with AccuPrime Taq, and panels B and C represent Platinum SuperMix replicates 1 and 2, respectively. The horizontal dashed line marks 100,000 on the log scaled y-axis on each plot.
(TIF)

Figure S2 **Comparison of quality control filtering between replicate 454 libraries created with different polymerases.** Each library was produced with the same set of DNA templates and barcoded primers. Libraries Platinum 1 and Platinum 2 where produced from the same amplification products but pooled and sequenced independently. The total number of raw sequences per library is given at the top of each bar. Note that the categories given in the key are in the same order as they appear in the plot; contaminants were present in all three libraries but accounted for <0.04% of each library.
(TIF)

Figure S3 **Bacterial operational taxonomic unit rarefaction curve for** ***Amblyomma americanum*** **tick DNA sample sets.** The shaded regions represent the conditional 95% confidence interval obtained from 1000 randomizations of the data.
(TIF)

Figure S4 **Non-metric multi-dimensional scaling (NMDS) of a Bray-Curtis distance matrix describing** ***Amblyomma americanum*** **bacterial communities.** Each of the 131 points symbolizes a single tick’s community; some points may overlap completely. Point and ellipse colors (red = Georgia ticks, black = archival) indicate the tick DNA collection of origin; ellipses represent 95% confidence intervals around group centroids. Non-overlapping centroids are considered significantly different at α = 0.05. The R^2^ value in the upper left corner of the plot describes the amount of variation in the data set explained by the groupings. The stress value is a measure of the disagreement between the rank order in the original data set and that in the NMDS (lower numbers indicate better agreement). Operational taxonomic units that differ in mean relative abundance between the groups are given in Table S4.
(TIF)

Figure S5 **Non-metric multi-dimensional scaling (NMDS) of a Jaccard distance matrix describing Georgian** ***Amblyomma americanum*** **bacterial communities.** Each point symbolizes a single tick’s community (n = 104); some points may overlap completely. Point and ellipse colors indicate life stage; ellipses represent 95% confidence intervals around life stage centroids. Non-overlapping centroids are considered significantly different at α = 0.05. R^2^ values in the upper left corner of plots describe the amount of variation in the data set explained by the groupings. The stress value is a measure of the disagreement between the rank order in the original data set and that in the NMDS (lower numbers indicate better agreement). Brown numbers indicate the species scores for select OTUs as follows: (1) *Coxiella*, (2) *Rickettsia*, (3) *Midichloria* 1, (4) *Borrelia*, (5) *Ehrlichia*, (6) Bacillales 1, (7) *Wolbachia*, (8) Rhizobiales 1, (9) *Rhodobaca*, (10) Bacillales 2, (11) *Nitriliruptor*, (12) Bacillales 3.
(TIF)

Figure S6 **Mean relative abundance of operational taxonomic units (OTUs) from Georgian ticks by life stage.** Significant differences (*p*<0.05) are labeled within rows by the letters that appear in cells. For example, within a row cells labeled A are significantly different from those labeled B or C but not cells labeled A. Empty cells do not differ from any group. Note the discontinuity between the white-black and yellow-red scales.
(TIF)

Figure S7 **Non-metric multi-dimensional scaling (NMDS) of a Jaccard distance matrix describing archival** ***Amblyomma americanum*** **bacterial communities.** Each point symbolizes a single tick’s community (n = 27); some points may overlap completely. Point and ellipse colors indicate life stage; ellipses represent 95% confidence intervals around life stage centroids. Non-overlapping centroids are considered significantly different at α = 0.05. R^2^ values in the upper left corner of plots describe the amount of variation in the data set explained by the groupings. The stress value given is a measure of the disagreement between the rank order in the original data set and that in the NMDS (lower numbers indicate better agreement). Numbers indicate the species scores for the plot as follows: (1) *Coxiella*, (2) *Rickettsia*, (3) *Midichloria* 1, (4) *Borrelia*, (5) *Francisella*, (6) *Midichloria* 2, (7) *Ehrlichia*, (8) Enterobacteriaceae, (9) *Pseudomonas* 1, (10) Burkholderiaceae, (11) Acidobacteria Gp1, (12) *Pseudomonas* 2, (13) Rhizobiales 2, (14) *Bacillus*, (15) Acetobacteraceae 1, (16) Acetobacteraceae 2, (17) Bacteria 1, (18) Bacteria 2.
(TIF)

Table S1 **Barcode sequences used to label eubacterial** ***rrs*** **gene variable regions 5–3 primers for sample multiplexing during pyrosequencing.**
(DOCX)

Table S2 **454 sequencing statistics from Titanium FLX plates.**
(DOCX)

Table S3 **Summary of previous reports of most abundant operational taxonomic units (OTUs) from** ***Amblyomma americanum*** **.**
(DOCX)

Table S4 **Metastats results comparing the differential abundance of bacterial operational taxonomic units between** ***Amblyomma americanum*** **DNA collections.**
(XLSX)
