# Supplementary figures and images for: Characterization of the Bacterial Communities of Life Stages of Free Living Lone Star Ticks (Amblyomma americanum)
Source: PLoS One. 2014 Jul 23;9(7):e102130. doi: 10.1371/journal.pone.0102130 (PMC4108322; doi:10.1371/journal.pone.0102130)

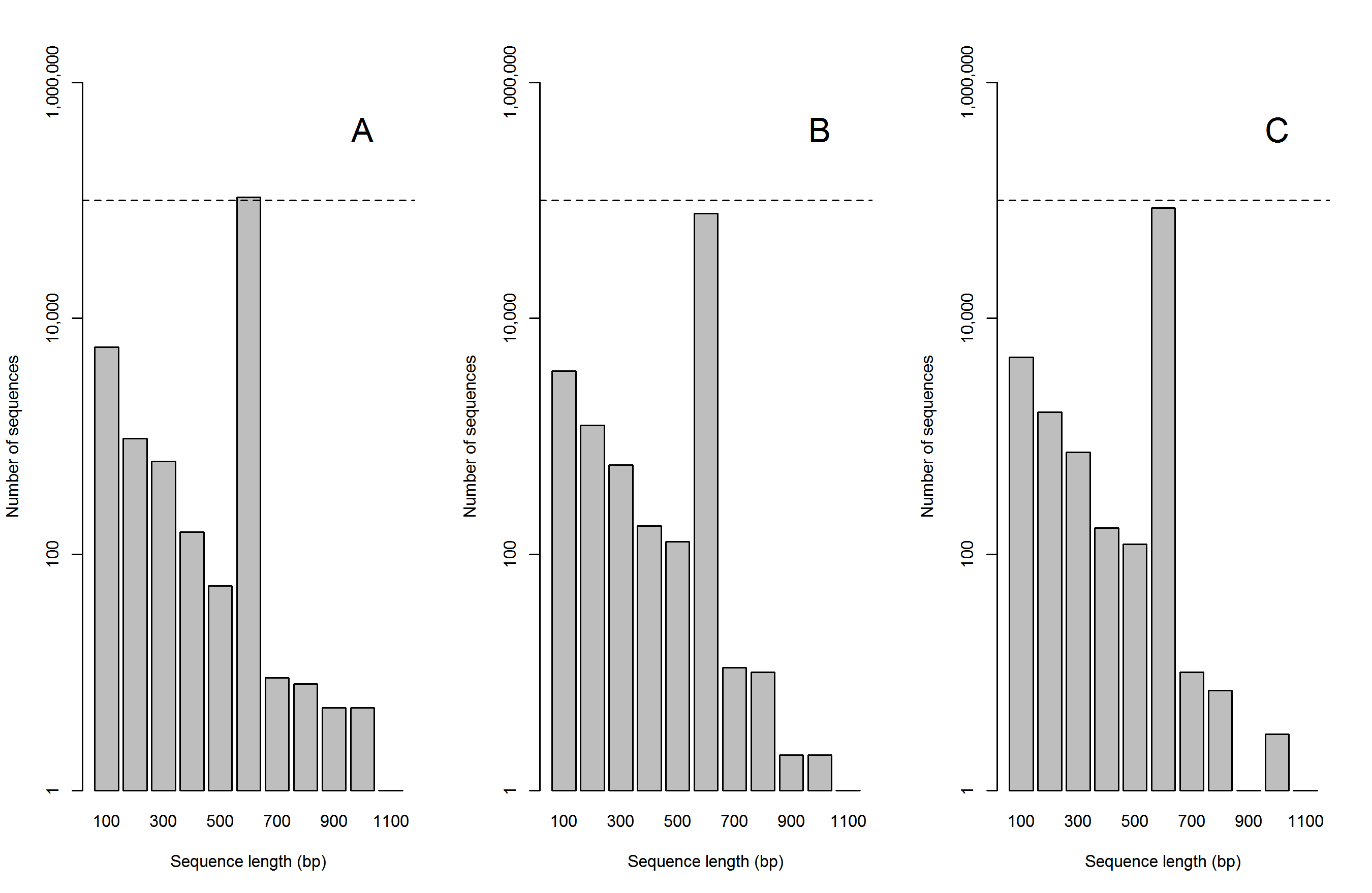

Supplement: Figure S1 — Comparison of raw 454 read length distributions between replicate libraries created with different polymerases. Each library was produced with the same set of DNA templates and barcoded primers. The polymerase varied between panels A and B/C, while B and C were produced from the same amplification products but pooled and sequenced independently. Panel A represents the library created with AccuPrime Taq, and panels B and C represent Platinum SuperMix replicates 1 and 2, respectively. The horizontal dashed line marks 100,000 on the log scaled y-axis on each plot. (TIF) [file pone.0102130.s001.tif]

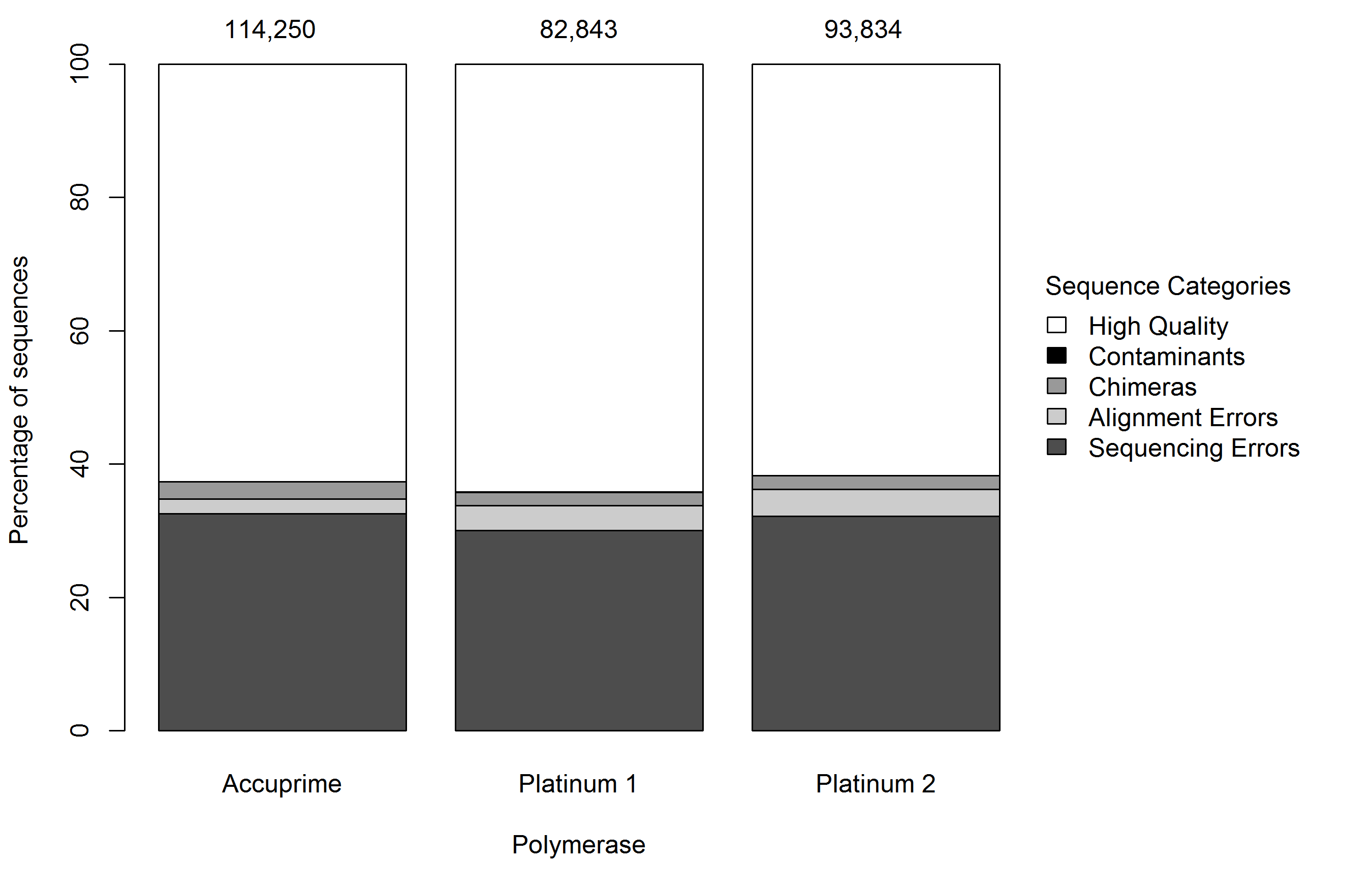

Supplement: Figure S2 — Comparison of quality control filtering between replicate 454 libraries created with different polymerases. Each library was produced with the same set of DNA templates and barcoded primers. Libraries Platinum 1 and Platinum 2 where produced from the same amplification products but pooled and sequenced independently. The total number of raw sequences per library is given at the top of each bar. Note that the categories given in the key are in the same order as they appear in the plot; contaminants were present in all three libraries but accounted for <0.04% of each library. (TIF) [file pone.0102130.s002.tif]

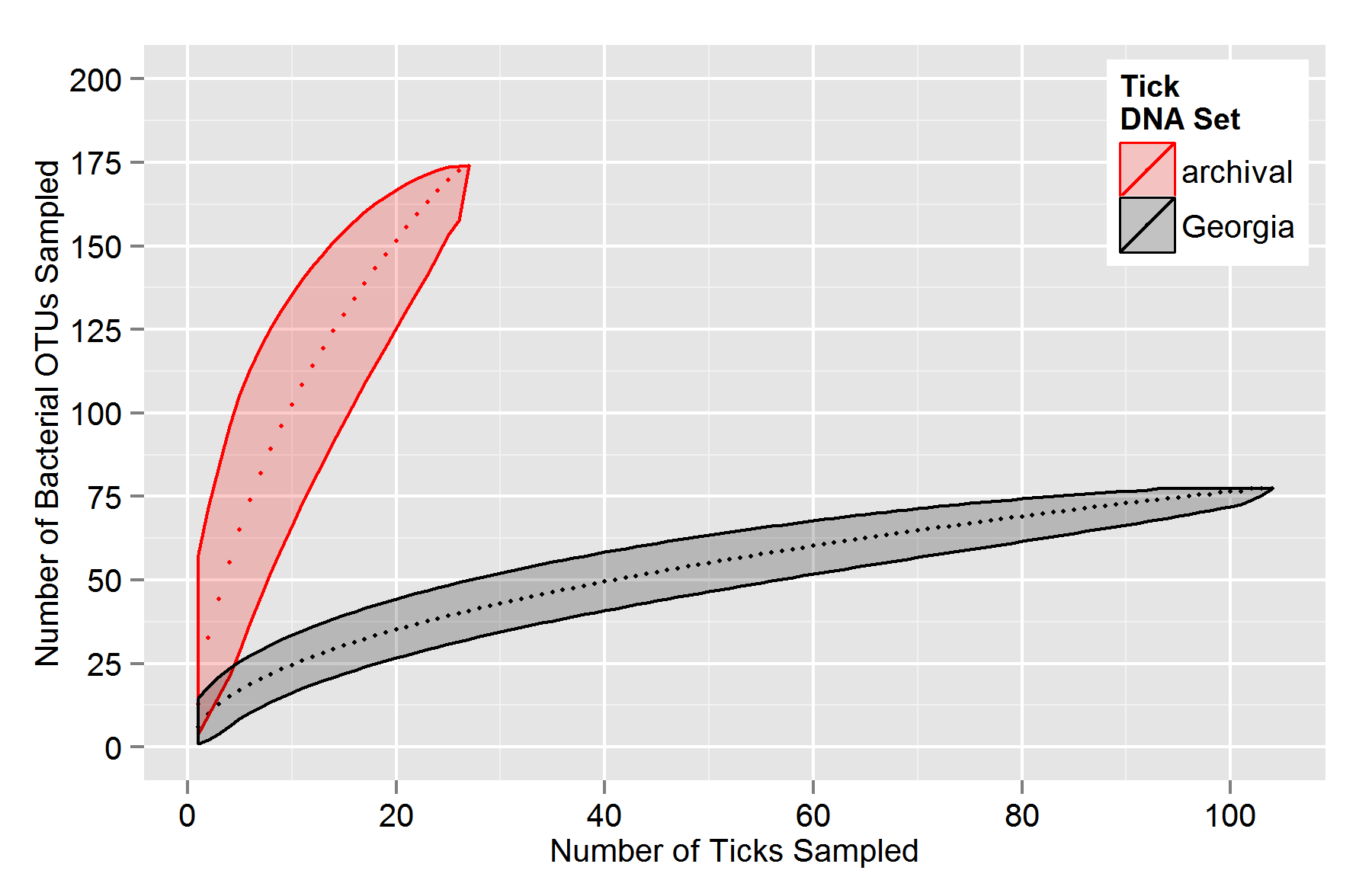

Supplement: Figure S3 — Bacterial operational taxonomic unit rarefaction curve for Amblyomma americanum tick DNA sample sets. The shaded regions represent the conditional 95% confidence interval obtained from 1000 randomizations of the data. (TIF) [file pone.0102130.s003.tif]

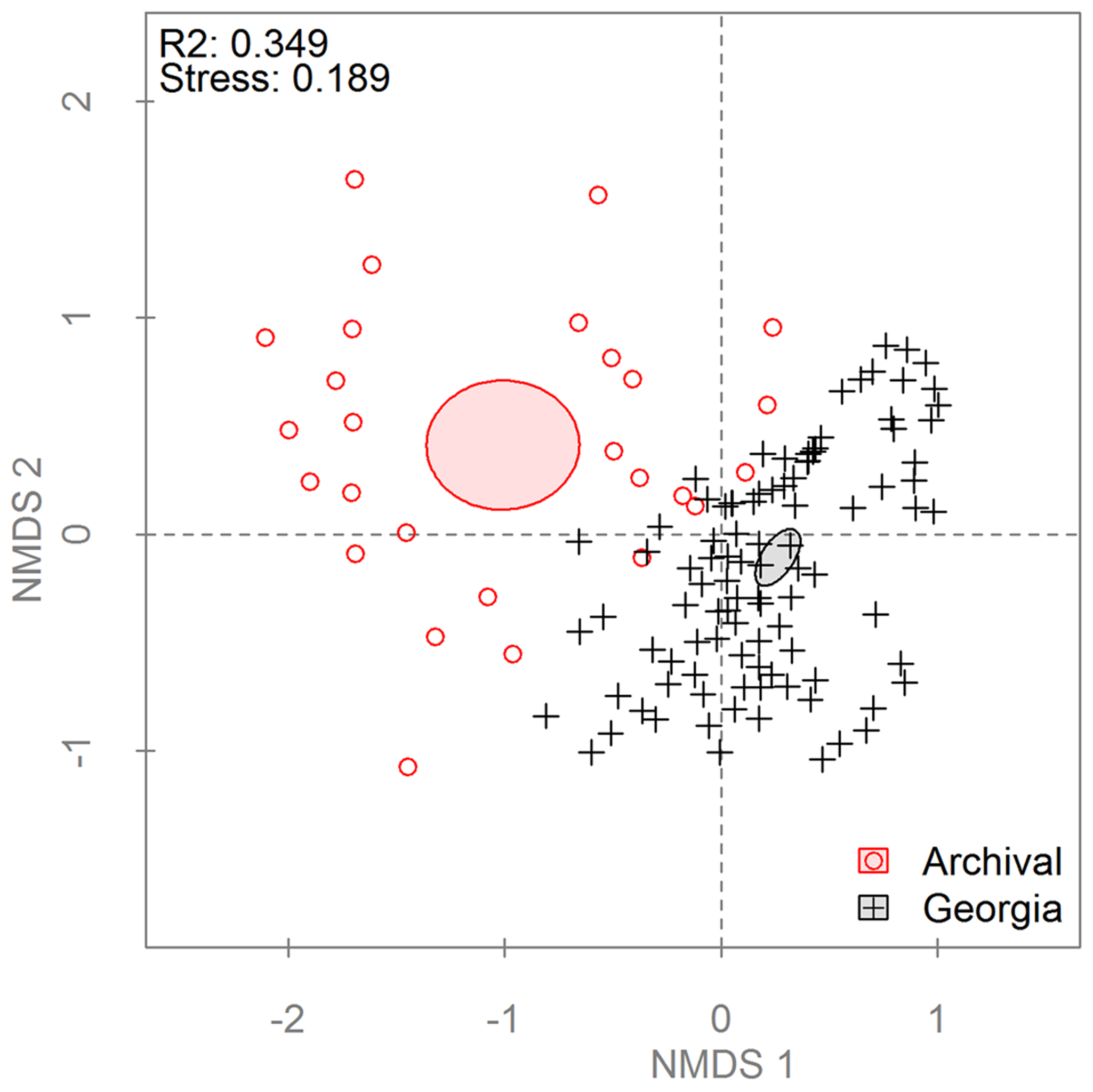

Supplement: Figure S4 — Non-metric multi-dimensional scaling (NMDS) of a Bray-Curtis distance matrix describing Amblyomma americanum bacterial communities. Each of the 131 points symbolizes a single tick’s community; some points may overlap completely. Point and ellipse colors (red = Georgia ticks, black = archival) indicate the tick DNA collection of origin; ellipses represent 95% confidence intervals around group centroids. Non-overlapping centroids are considered significantly different at α = 0.05. The R2 value in the upper left corner of the plot describes the amount of variation in the data set explained by the groupings. The stress value is a measure of the disagreement between the rank order in the original data set and that in the NMDS (lower numbers indicate better agreement). Operational taxonomic units that differ in mean relative abundance between the groups are given in Table S4. (TIF) [file pone.0102130.s004.tif]

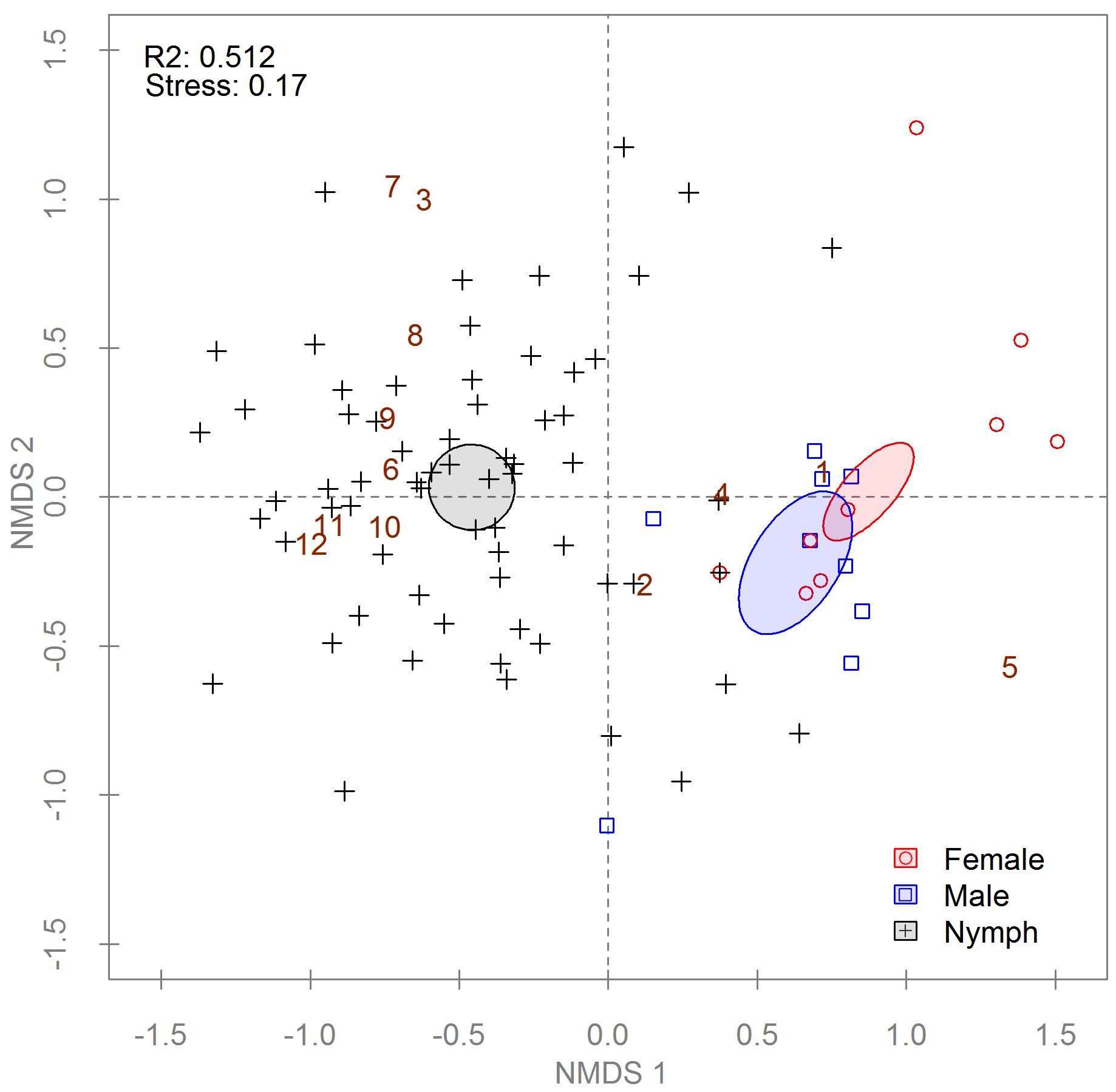

Supplement: Figure S5 — Non-metric multi-dimensional scaling (NMDS) of a Jaccard distance matrix describing Georgian Amblyomma americanum bacterial communities. Each point symbolizes a single tick’s community (n = 104); some points may overlap completely. Point and ellipse colors indicate life stage; ellipses represent 95% confidence intervals around life stage centroids. Non-overlapping centroids are considered significantly different at α = 0.05. R2 values in the upper left corner of plots describe the amount of variation in the data set explained by the groupings. The stress value is a measure of the disagreement between the rank order in the original data set and that in the NMDS (lower numbers indicate better agreement). Brown numbers indicate the species scores for select OTUs as follows: (1) Coxiella, (2) Rickettsia, (3) Midichloria 1, (4) Borrelia, (5) Ehrlichia, (6) Bacillales 1, (7) Wolbachia, (8) Rhizobiales 1, (9) Rhodobaca, (10) Bacillales 2, (11) Nitriliruptor, (12) Bacillales 3. (TIF) [file pone.0102130.s005.tif]

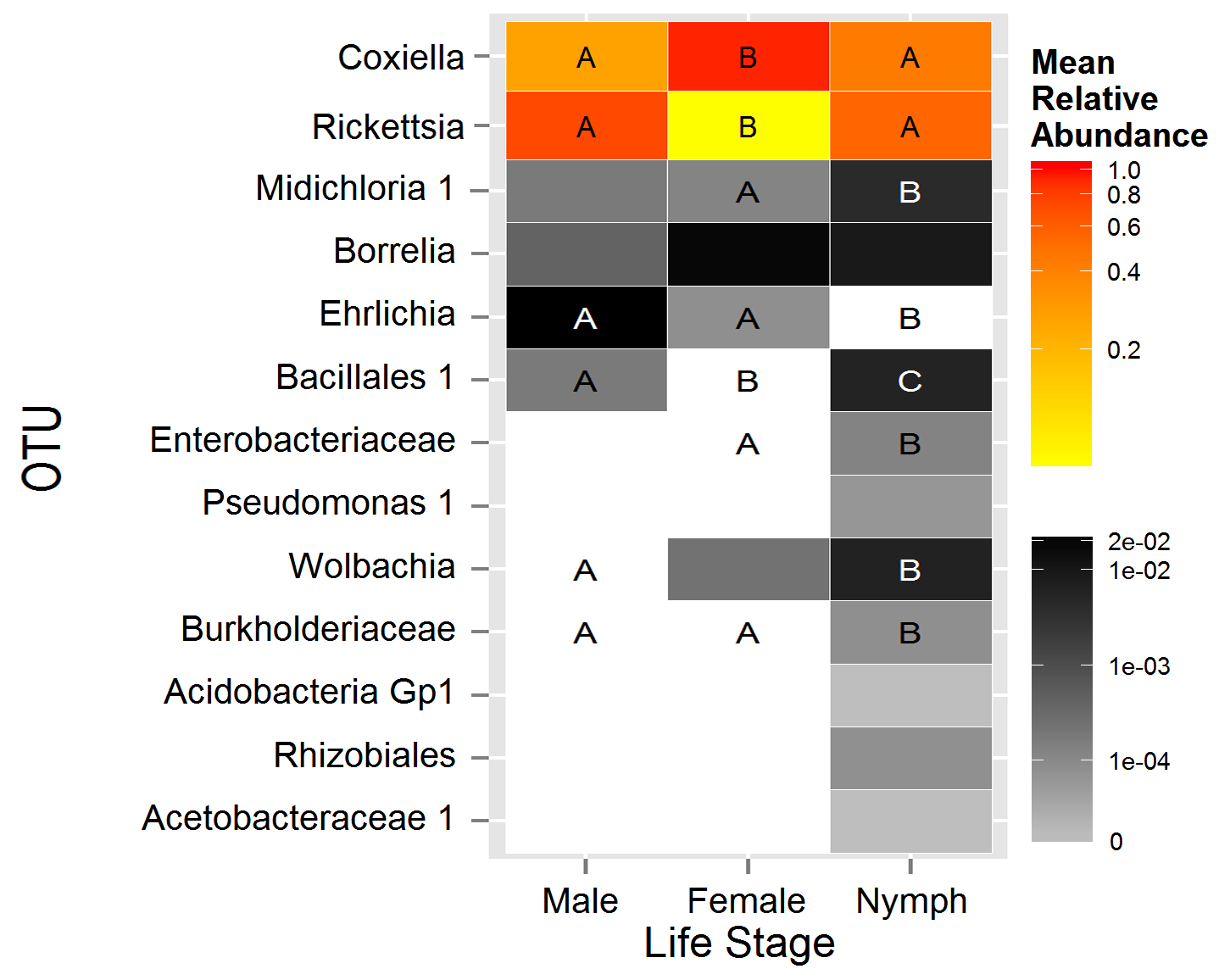

Supplement: Figure S6 — Mean relative abundance of operational taxonomic units (OTUs) from Georgian ticks by life stage. Significant differences (p<0.05) are labeled within rows by the letters that appear in cells. For example, within a row cells labeled A are significantly different from those labeled B or C but not cells labeled A. Empty cells do not differ from any group. Note the discontinuity between the white-black and yellow-red scales. (TIF) [file pone.0102130.s006.tif]

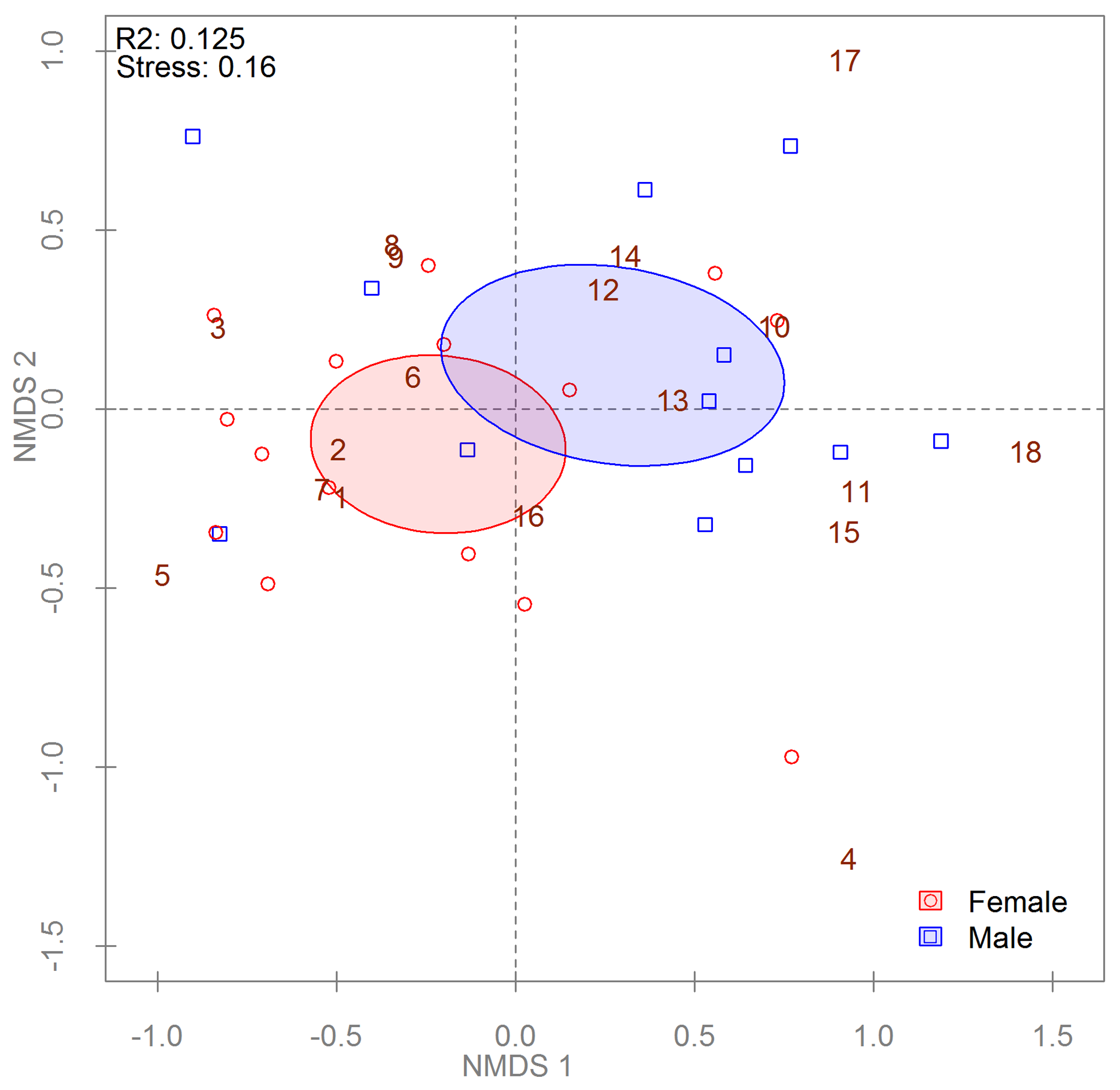

Supplement: Figure S7 — Non-metric multi-dimensional scaling (NMDS) of a Jaccard distance matrix describing archival Amblyomma americanum bacterial communities. Each point symbolizes a single tick’s community (n = 27); some points may overlap completely. Point and ellipse colors indicate life stage; ellipses represent 95% confidence intervals around life stage centroids. Non-overlapping centroids are considered significantly different at α = 0.05. R2 values in the upper left corner of plots describe the amount of variation in the data set explained by the groupings. The stress value given is a measure of the disagreement between the rank order in the original data set and that in the NMDS (lower numbers indicate better agreement). Numbers indicate the species scores for the plot as follows: (1) Coxiella, (2) Rickettsia, (3) Midichloria 1, (4) Borrelia, (5) Francisella, (6) Midichloria 2, (7) Ehrlichia, (8) Enterobacteriaceae, (9) Pseudomonas 1, (10) Burkholderiaceae, (11) Acidobacteria Gp1, (12) Pseudomonas 2, (13) Rhizobiales 2, (14) Bacillus, (15) Acetobacteraceae 1, (16) Acetobacteraceae 2, (17) Bacteria 1, (18) Bacteria 2. (TIF) [file pone.0102130.s007.tif]
