# Supplementary material for: Characterization of the Bacterial Communities of Life Stages of Free Living Lone Star Ticks (Amblyomma americanum)
Source: PLoS One. 2014 Jul 23;9(7):e102130. doi: 10.1371/journal.pone.0102130 (PMC4108322; doi:10.1371/journal.pone.0102130)
Supplement: Table S1 — Barcode sequences used to label eubacterial rrs gene variable regions 5–3 primers for sample multiplexing during pyrosequencing. (DOCX) [file pone.0102130.s008.docx]

**Table S1. Barcode sequences used to label eubacterial *rrs* gene** **variable regions 5-3 primers for sample multiplexing during pyrosequencing.**

| **Barcode Name** | **Barcode Sequence** |
| --- | --- |
| v2bBar8L | CACGC |
| v2bBar23L | CGCAAC |
| v2bBar174L | TGAAGC |
| v2bBar212L | TCACAC |
| v2bBar622L | ACGCGC |
| v2bBar72L | CCTCTC |
| v2bBar559L | AGACAC |
| v2bBar31L | CGACTC |
| v2bBar551L | AGCTTC |
| v2bBar1149L | AAGCCGC |
| v2bBar15L | CAAGAAC |
| v2bBar556L | AGTTGGC |
| v2bBar144L | TATCAAC |
| v2bBar575L | AGGCGGC |
| v2bBar48L | CGGTATC |
| v2bBar741L | ATACCAC |
| v2bBar228L | TCGCGGC |
| v2bBar807L | ATCTTAC |
| v2bBar1273L | AACCAGC |
| v2bBar441L | TTCGAGC |
| v2bBar1174L | AAGGTGC |
| v2bBar209L | TCTTGGC |
| v2bBar153L | TAATCTC |
| v2bBar213L | TCACCTC |
| v2bBar146L | TATTGAC |
| v2bBar554L | AGTCGAC |
| v2bBar646L | ACGGCTC |
| v2bBar158L | TGCGTTC |
| v2bBar207L | TCTCGAC |
| v2bBar601L | ACTCCTC |
| v2bBar419L | TTCATAC |
| v2bBar26L | CGTCGTC |
| v2bBar159L | TGCCGAAC |
| v2bBar147L | TATTCGTC |
| v2bBar141L | TAGGAATC |
| v2bBar119L | CCGGCCAC |
| v2bBar1267L | AACCTGGC |
| v2bBar637L | ACGAAGTC |
| v2bBar435L | TTCGTGGC |
| v2bBar433L | TTCGCGAC |
| v2bBar1156L | AAGAGTTC |
| v2bBar1173L | AAGGCCTC |
| v2bBar599L | ACTAATTC |
| v2bBar393L | TTGGAGGC |
| v2bBar350L | TTATCGGC |
| v2bBar1196L | AACTGTTC |
| v2bBar1031L | ATTCGTAC |
| v2bBar76L | CCTTCCGC |
| v2bBar1225L | AACGAGGC |
| v2bBar236L | TCGAGGAAC |
| v2bBar731L | ACCGGAAGC |
| v2bBar1250L | AACGGAGTC |
| v2bBar187L | TGGTTGGTC |
| v2bBar162L | TGTCCGGTC |
| v2bBar1292L | AACCGTGTC |
