# Supplementary material for: Characterization of the Bacterial Communities of Life Stages of Free Living Lone Star Ticks (Amblyomma americanum)
Source: PLoS One. 2014 Jul 23;9(7):e102130. doi: 10.1371/journal.pone.0102130 (PMC4108322; doi:10.1371/journal.pone.0102130)
Supplement: Table S2 — 454 sequencing statistics from Titanium FLX plates. (DOCX) [file pone.0102130.s009.docx]

Table S1. 454 sequencing statistics from Titanium FLX plates.

| **Plate ID** | **Plate Quarter ID** | **Polymerase*** | **Tick DNA Set** | **Experiment** | **Total No. DNAs** | **No. Raw Reads** | **No. High Quality Reads (% Raw)** | **No. Tick DNAs Retained^α^** | **No. Reads in Analysis^β^** |
| --- | --- | --- | --- | --- | --- | --- | --- | --- | --- |
| 1 | A | AccuPrime | Archival | Life stage | 50 | 114,250 | 85,465 (75%) | 27 | 59,028 |
|  |  |  |  | Polymerase | 50 | 114,250 | 71,552 (63%) | 26 | 64,411 |
| 1 | B | Platinum | Archival | Polymerase | 50 | 82,843 | 53,175 (64%) | 26 | 49,269 |
| 1 | C | Platinum | Archival | Polymerase | 50 | 93,834 | 57,912 (62%) | 26 | 53,718 |
| 2 | A | AccuPrime | Georgia | Life stage | 50 | 82,087 | 55,560 (68%) | 22 | 45,579 |
| 2 | B | AccuPrime | Georgia | Life stage | 50 | 71,821 | 47,664 (66%) | 19 | 33,604 |
| 2 | C | AccuPrime | Georgia | Life stage | 50 | 64,743 | 36,245 (56%) | 15 | 22,875 |
| 2 | D | AccuPrime | Georgia | Life stage | 50 | 60,428 | 44,004 (73%) | 15 | 28,585 |
| 3 | A | Platinum | Georgia | Life stage | 50 | 276,596 | 177,476 (64%) | 33 | 160,830 |

*Polymerases used include AccuPrime Taq High Fidelity and Platinum PCR SuperMix High Fidelity (Invitrogen, Carlsbad, California, USA).

^α^ Discarded DNAs were inferior replicates of retained DNAs, were not sequenced to a depth of at least 1000 sequences, or belonged to groups that were insufficiently sampled to be included in the analysis.

^β^ Total number of high quality sequences from retained ticks. Includes singleton OTUs.
