# Supplementary material for: Characterization of the Bacterial Communities of Life Stages of Free Living Lone Star Ticks (Amblyomma americanum)
Source: PLoS One. 2014 Jul 23;9(7):e102130. doi: 10.1371/journal.pone.0102130 (PMC4108322; doi:10.1371/journal.pone.0102130)
Supplement: Table S3 — Summary of previous reports of most abundant operational taxonomic units (OTUs) from Amblyomma americanum . (DOCX) [file pone.0102130.s010.docx]

Table S3. Summary of previous reports of most abundant operational taxonomic units (OTUs) from *Amblyomma americanum*.

| OTU | Classification  (Class) | GenBank ID | Closest GenBank BLAST Hit  (GenBank ID, % identity) | Previous Reports from Ticks? | Notes |
| --- | --- | --- | --- | --- | --- |
| 1 | Genus *Coxiella* (Gammaproteobacteria) | KJ130495 | *Coxiella* endosymbiont of *A. americanum*  (AY939824.1, 99%) | *A. americanum* [1] | Considered primary symbiont of *A. americanum* [2]. Related symbionts found in many other tick species [reviewed in 3]. |
| 2 | Genus *Rickettsia* (Alphaproteobacteria) | KJ130496 | *R. amblyommii*  (NR_074471.1, 99%) | *A. americanum* [4]  Other *Amblyomma*, eg. *A. cajennense* and *A. coelebs* [5] | Many additional citations exist; some are given in the main article. |
| 3 | Genus *Midichloria* 1 (Alphaproteobacteria) | KJ130497 | *M. mitochondrii* strain AamerB  (JQ678691.1, 100% ) | *A. americanum* [6]  *A. triste* [7]  *A. tuberculatum* [8]  *D. andersoni* [9]  *Ha. punctate* [8]  *Hy. marginatum* [8]  *Hy. truncatum* [8]  *I. ricinus* [8]  *I. uriae* [8]  *Rh. turanicus* [8]  *Rh. bursa* [8] | A likely facultative symbiont [10]. |
| 4 | Genus *Borrelia*  (Spirochaetes) | KJ130498 | *B. lonestari*  (AY166715.1, 100%) | *A. americanum* [11]  *C. capensis* [12] | *C. capensis*’s *Borrelia* has not been definitively identified as *B. lonestari*, but available evidence supports this identification. |
| 5 | Genus *Francisella* (Gammaproteobacteria) | KJ130499 | *Francisella* spp. both pathogenic and symbiotic (e.g. JQ764629.1, 100%) | *A. americanum* [reviewed in 13]  *A. maculatum* [14]  *Dermacentor* spp. [reviewed in 3]  *Hy. marginatum* [15] | Widespread symbiont of many tick species, but *A. americanum* is not known to carry a symbiotic form. |
| 6 | Genus *Midichloria* 2 (Alphaproteobacteria) | KJ130500 | *M. mitochondrii* strain AamerC  (JQ678692.1, 100%) | *A. americanum* [6]  *A. triste* [7]  *A. tuberculatum* [8]  *D. andersoni* [9]  *Ha. punctate* [8]  *Hy. marginatum* [8]  *Hy. truncatum* [8]  *I. ricinus* [8]  *I. uriae* [8]  *Rh. turanicus* [8]  *Rh. bursa* [8] | A likely facultative symbiont [10]. |
| 7 | Genus *Ehrlichia* (Alphaproteobacteria) | KJ130501 | *Ehrlichia* spp.  (e.g. U96436.1, 100%) | *A. americanum* [reviewed in 13]  *D. variabilis* [16]  *Rh. sanguineus* [17] |  |
| 8 | Order Bacillales 1  (Bacilli) | KJ130502 | *Bacillus* spp.  (e.g. EU530599.1, 100%) | *A. americanum* [18,19]  *A. testudinarium* [20]  *Ha. formosensis* [20]  *I. ovatus* [20]  *I. persulcatus* [20]  *I. ricinus* [20,21]  *I. scapularis* [19]  *Rh. microplus* [22] | Common environmental bacteria, but also known symbionts of other arthropods. Notably *Bacillus* protects against fungal pathogens in bees [reviewed in 23]. |
| 9 | Family Enterobacteriaceae (Gammaproteobacteria) | KJ130503 | Many genera; 60% of sequences classified to *Yersinia*  (e.g. BX936398.1, 100%) | *A. americanum* [18,19,24]  *I. scapularis* [19] | Many insect symbionts belong to this family (e.g. *Sodalis* [25], *Wigglesworthia* [26], *Buchnera* [27]), but it also contains common and diverse environmental bacteria [28] and pathogens [29]. |
| 10 | Genus *Pseudomonas* 1  (Gammaproteobacteria) | KJ130504 | *Pseudomonas* spp.  (e.g. KF147045.1, 100%) | *A. americanum* [18,24]  *I. ricinus* [21]  *Rh. microplus* [22] | Common environmental bacteria. Demonstrated *in vitro* lysis of vector-borne parasites by some species [30] |
| 11 | Genus *Wolbachia* (Alphaproteobacteria) | KJ130505 | *Wolbachia* strains  (e.g. KC522606.1, 99%) | *A. americanum* [31]  *I. ricinus* [21,32]  *I. scapularis* [33]  *Rh. microplus* [22]  *Rh. sanguineus* [34] | Widespread symbiont of many arthropods known for reproductive manipulation of hosts and protective abilities [reviewed in 35]. |
| 12 | Order Rhizobiales 1  (Alphaproteobacteria) | KJ130506 | Many genera; 52% of sequences classified to Bradyrhizobiaceae  (e.g. GU125653.2, 99%) | *A. americanum* [36]  *I. ricinus* [21] | Common nitrogen-fixing plant symbionts [reviewed in 37], but also found in association with ants [38,39]. |
| 13 | Family Burkholderiaceae (Betaproteobacteria) | KJ130507 | Many genera; 78% of sequences classified to *Burkholderia*  (e.g. KF059270.1, 100%) | *A. americanum* [19,24]  *I. ricinus* [21]  *I. scapularis* [19,40] | *Burkholderia* exist as free-living [41], plant symbionts [42], arthropod symbionts [43], and vertebrate pathogens [44]. |
| 14 | Family “Gp1” *incertae sedis*  (Acidobacteria) | KJ130508 | Unidentified clones  (e.g. HQ598755.1, 100%) | *A. variegatum* [20] | “Gp1” bacteria are iron oxidizers previously found in environmental samples, like iron-rich ground water [45]. |
| 15 | Genus *Rhodobaca* (Alphaproteobacteria) | KJ130509 | Unidentified clones  (e.g. AB297418.1, 100%) | No | Genus containing anoxygenic purple nonsulfur bacteria usually associated with lake waters and soil [46]. Never previously reported in association with ixodid ticks, but its closest phylogenetic relatives in the genus *Rhodobacter* have been (*I. scapularis* [19], *Rh. microplus* [22], and *I. ricinus* [21]). |
| 16 | Order Bacillales 2  (Bacilli) | KJ130510 | Unidentified clones  (e.g. JF968215.1, 100%) | *A. americanum* [18,19]  *A. testudinarium* [20]  *Ha. formosensis* [20]  *I. ovatus* [20]  *I. persulcatus* [20]  *I. ricinus* [20,21]  *I. scapularis* [19]  *Rh. microplus* [22] | Common environmental bacteria, but also known symbionts of other arthropods. Notably *Bacillus* protects against fungal pathogens in bees [reviewed in 23]. |
| 17 | Genus *Pseudomonas* 2 (Gammaproteobacteria) | KJ130511 | *Pseudomonas* spp.  (e.g. GQ232466.1, 100%) | *A. americanum* [18,24]  *I. ricinus* [21]  *Rh. microplus* [22] | Common environmental bacteria. Demonstrated in vitro lysis of vector-borne parasites by some species [30]. |
| 18 | Genus *Nitriliruptor* (Actinobacteria) | KJ130512 | *N. alkaliphilus*  (NR_044203.1, 100%) | References to unclassified Actinobacteria:  *A. americanum* [18,19]  *A. variegatum* [20]  *D. variabilis* [40]  *I. scapularis* [21,40] | *Nitriliruptor* has been previously documented from soil samples [47,48]. Actinobacteria are common environmental bacteria and eukaryote-associates [reviewed in 49]. |
| 19 | Genus *Methylobacterium* (Alphaproteobacteria) | KJ130513 | *Methylobacterium* spp.  (e.g. AB698724.1, 100%) | *A. americanum* [18]  *I. ricinus* [21] | Common environmental bacteria associated with soil and plants [e.g. 50]. It is known from the human mouth [51] and skin [52] microbiome and may also be pathogenic [53]. |
| 20 | Order Rhizobiales 2 (Alphaproteobacteria) | KJ130514 | Unidentified clones  (e.g. JQ047240.1, 100%) | *A. americanum* [24]  *D. variabilis* [40]  *I. ricinus* [21]  *I. scapularis* [40]  *Rh. microplus* [22] | Common nitrogen-fixing plant symbionts [reviewed in 37], but also found in association with ants [38,39]. |
| 21 | Family Sphingomonadaceae (Alphaproteobacteria) | KJ130515 | *Sphingomonas* spp.; 97% of sequences classified to *Sphingomonas*  (e.g. KF542913.1, 100%) | *A. americanum* [19,36]  *I. ricinus* [21]  *I. scapularis* [19] | *Sphingomonas* spp. are known arthropod associates, including mosquitoes (*Aedes aegypti*) [54] and termites (*Macrotermes natalensis* and *Nasutitermes* sp.) [55]. |
| 22 | Order Bacillales 3  (Bacilli) | KJ130516 | Unidentified clones  (e.g. AB637120.1, 100%) | *A. americanum* [18,19]  *A. testudinarium* [20]  *Ha. formosensis* [20]  *I. ovatus* [20]  *I. persulcatus* [20]  *I. ricinus* [20,21]  *I. scapularis* [19]  *Rh. microplus* [22] | Common environmental bacteria, but also known symbionts of other arthropods. Notably *Bacillus* protects against fungal pathogens in bees [reviewed in 23]. |
| 23 | Order Actinomycetales (Actinobacteria) | KJ130517 | *Mycobacterium* spp.; 99% of sequences classified to *Mycobacterium*  (e.g. KF224994.1, 100%) | *A. americanum* [19]  *D. variabilis* [40]  *I. ricinus* [21]  *I. scapularis* [19] | Common environmental bacteria (soil, water). Also common pathogens of wildlife (birds, mammals) [56]. |

The OTU number ranks the groups in order of decreasing abundance. In the classification column, the lowest taxon that all sequences within the OTU could be assigned to is provided first, followed by the class assignment in parentheses. Numbers are added to this column to differentiate OTUs classified to otherwise identical taxons; these are the same numbers used in the main paper. GenBank IDs are given for a representative sequence from each OTU, which was defined as the sequence with the smallest maximum distance from all other sequences in the OTU. The closest GenBank BLAST Hit was found by comparing the representative sequence against the GenBank database and reporting the hit with the highest identity. If there was no single best hit, this is indicated by the example designation before the GenBank ID number. The fifth column contains references for tick species from which these OTUs have previously been reported and is not exhaustive. Tick genus abbreviations are as follows: *A. Amblyomma, C. Carios, D. Dermacentor, Ha. Haemaphysalis, Hy. Hyalomma, I. Ixodes,* and *Rh. Rhipicephalus*.

References

1. Jasinskas A, Zhong J, Barbour AG (2007) Highly prevalent *Coxiella* sp. bacterium in the tick vector *Amblyomma americanum*. Appl Environ Microbiol 73: 334–336. doi:10.1128/aem.02009-06.

2. Zhong JM, Jasinskas A, Barbour AG (2007) Antibiotic treatment of the tick vector *Amblyomma americanum* reduced reproductive fitness. PLoS One 2: e405. doi:10.1371/journal.pone.0000405.

3. Ahantarig A, Trinachartvanit W, Baimai V, Grubhoffer L (2013) Hard ticks and their bacterial endosymbionts (or would be pathogens). Folia Microbiol (Praha): 1–10. doi:10.1007/s12223-013-0222-1.

4. Burgdorfer W, Hayes SF, Thomas L, Lancaster J (1981) A new spotted fever group rickettsia from the lone star tick, *Amblyomma americanum*. In: Burgdorfer W, Anacker W, editors. Rickettsiae and Rickettsial Diseases. New York: Academic Press. pp. 595–602.

5. Labruna MB, Whitworth T, Bouyer DH, McBride J, Camargo LMA, et al. (2004) *Rickettsia bellii* and *Rickettsia amblyommii* in *Amblyomma* ticks from the State of Rondônia, Western Amazon, Brazil. J Med Entomol 41: 1073–1081.

6. Williams-Newkirk AJ, Rowe LA, Mixson-Hayden TR, Dasch GA (2012) Presence, genetic variability, and potential significance of “*Candidatus* Midichloria mitochondrii” in the lone star tick *Amblyomma americanum*. Exp Appl Acarol 58: 291–300. doi:10.1007/s10493-012-9582-5.

7. Venzal J, Estrada-Peña A, Portillo A, Mangold A, Castro O, et al. (2008) Detection of alpha and gamma-proteobacteria in *Amblyomma triste* (Acari: Ixodidae) from Uruguay. Exp Appl Acarol 44: 49–56.

8. Epis S, Sassera D, Beninati T, Lo N, Beati L, et al. (2008) *Midichloria mitochondrii* is widespread in hard ticks (Ixodidae) and resides in the mitochondria of phylogenetically diverse species. Parasitology 135: 485–494. doi:doi:10.1017/S0031182007004052.

9. Dergousoff SJ, Chilton NB (2011) Novel genotypes of *Anaplasma bovis*, “*Candidatus* Midichloria” sp. and *Ignatzschineria* sp. in the Rocky Mountain wood tick, *Dermacentor andersoni*. Vet Microbiol 150: 100–106. doi:10.1016/j.vetmic.2011.01.018.

10. Pistone D, Sacchi L, Lo N, Epis S, Pajoro M, et al. (2012) *Candidatus* Midichloria mitochondrii: symbiont or parasite of tick mitochondrii? In: Zchori-Fein E, Bourtzis K, editors. Manipulative Tenants: Bacteria Associated With Arthropods. Frontiers in Microbiology. Boca Raton: CRC Press. pp. 113–126.

11. Barbour AG, Maupin GO, Teltow GJ, Carter CJ, Piesman J (1996) Identification of an uncultivable *Borrelia* species in the hard tick *Amblyomma americanum*: possible agent of a Lyme disease-like illness. J Infect Dis 173: 403–409.

12. Reeves W, Loftis A, Sanders F, Spinks M, Wills W, et al. (2006) *Borrelia*, *Coxiella*, and *Rickettsia* in *Carios capensis* (Acari: Argasidae) from a brown pelican (*Pelecanus occidentalis*) rookery in South Carolina, USA. Exp Appl Acarol 39: 321–329.

13. Childs JE, Paddock CD (2003) The ascendancy of *Amblyomma americanum* as a vector of pathogens affecting humans in the United States. Annu Rev Entomol 48: 307–337. doi:10.1146/annurev.ento.48.091801.112728.

14. Scoles GA (2004) Phylogenetic analysis of the *Francisella*-like endosymbionts of *Dermacentor* ticks. J Med Entomol 41: 277–286.

15. Montagna M, Chouaia B, Pella F, Mariconti M, Pistone D, et al. (2012) Screening for bacterial DNA in the hard tick *Hyalomma marginatum* (Ixodidae) from Socotra Island (Yemen): detection of *Francisella*-like endosymbiont. J Entomol Acarol Res 44. Available: http://www.pagepressjournals.org/index.php/jear/article/view/502. Accessed 7 January 2013.

16. Johnson EM, Ewing SA, Barker RW, Fox JC, Crow DW, et al. (1998) Experimental transmission of *Ehrlichia canis* (Rickettsiales: Ehrlichieae) by *Dermacentor variabilis* (Acari: Ixodidae). Vet Parasitol 74: 277–288. doi:10.1016/S0304-4017(97)00073-3.

17. Groves MG, Dennis GL, Amyx HL, Huxsoll DL (1975) Transmission of *Ehrlichia canis* to dogs by ticks (*Rhipicephalus sanguineus*). Am J Vet Res 36: 937–940.

18. Heise SR, Elshahed MS, Little SE (2010) Bacterial diversity in *Amblyomma americanum* (Acari: Ixodidae) with a focus on members of the genus *Rickettsia*. J Med Entomol 47: 258–268. doi:10.1603/me09197.

19. Yuan D (2010) A metagenomic study of the tick midgut. MS Thesis, The University of Texas. Available: http://digitalcommons.library.tmc.edu/utgsbs_dissertations/85. Accessed 17 July 2013.

20. Nakao R, Abe T, Nijhof AM, Yamamoto S, Jongejan F, et al. (2013) A novel approach, based on BLSOMs (Batch Learning Self-Organizing Maps), to the microbiome analysis of ticks. ISME J. Available: http://www.nature.com/doifinder/10.1038/ismej.2012.171. Accessed 28 March 2013.

21. Carpi G, Cagnacci F, Wittekindt NE, Zhao F, Qi J, et al. (2011) Metagenomic profile of the bacterial communities associated with *Ixodes ricinus* ticks. PLoS ONE 6: e25604. doi:10.1371/journal.pone.0025604.

22. Andreotti R, León AAP de, Dowd SE, Guerrero FD, Bendele KG, et al. (2011) Assessment of bacterial diversity in the cattle tick *Rhipicephalus* (*Boophilus*) *microplus* through tag-encoded pyrosequencing. BMC Microbiol 11: 6. doi:10.1186/1471-2180-11-6.

23. Kaltenpoth M, Engl T (2013) Defensive microbial symbionts in Hymenoptera. Funct Ecol epub online ahead of print: 1–13. doi:10.1111/1365-2435.12089.

24. Clay K, Klyachko O, Grindle N, Civitello D, Oleske D, et al. (2008) Microbial communities and interactions in the lone star tick, *Amblyomma americanum*. Mol Ecol 17: 4371–4381. doi:10.1111/j.1365-294X.2008.03914.x.

25. Dale C, Maudlin I (1999) *Sodalis* gen. nov. and *Sodalis glossinidius* sp. nov., a microaerophilic secondary endosymbiont of the tsetse fly *Glossina morsitans morsitans*. Int J Syst Bacteriol 49: 267–275. doi:10.1099/00207713-49-1-267.

26. Pais R, Lohs C, Wu Y, Wang J, Aksoy S (2008) The obligate mutualist *Wigglesworthia glossinidia* influences reproduction, digestion, and immunity processes of its host, the tsetse fly. Appl Environ Microbiol 74: 5965–5974. doi:10.1128/AEM.00741-08.

27. Douglas AE (1998) Nutritional interactions in insect-microbial symbioses: aphids and their symbiotic bacteria *Buchnera*. Annu Rev Entomol 43: 17–37. doi:10.1146/annurev.ento.43.1.17.

28. Degelmann DM, Kolb S, Dumont M, Murrell JC, Drake HL (2009) Enterobacteriaceae facilitate the anaerobic degradation of glucose by a forest soil. FEMS Microbiol Ecol 68: 312–319. doi:10.1111/j.1574-6941.2009.00681.x.

29. Perry RD, Fetherston JD (1997) *Yersinia pestis*--etiologic agent of plague. Clin Microbiol Rev 10: 35–66.

30. Mercado TI, Colon-Whitt A (1982) Lysis of *Trypanosoma cruzi* by *Pseudomonas fluorescens*. Antimicrob Agents Chemother 22: 1051–1057.

31. Zhang X, Norris DE, Rasgon JL (2011) Distribution and molecular characterization of *Wolbachia* endosymbionts and filarial nematodes in Maryland populations of the lone star tick (*Amblyomma americanum*). FEMS Microbiol Ecol 77: 50–56. doi:10.1111/j.1574-6941.2011.01089.x.

32. Plantard O, Bouju-Albert A, Malard M-A, Hermouet A, Capron G, et al. (2012) Detection of *Wolbachia* in the tick *Ixodes ricinus* is due to the presence of the hymenoptera endoparasitoid *Ixodiphagus hookeri*. PLoS ONE 7: e30692. doi:10.1371/journal.pone.0030692.

33. Benson MJ, Gawronski JD, Eveleigh DE, Benson DR (2004) Intracellular symbionts and other bacteria associated with deer ticks (*Ixodes scapularis*) from Nantucket and Wellfleet, Cape Cod, Massachusetts. Appl Environ Microbiol 70: 616–620. doi:10.1128/aem.70.1.616-620.2004.

34. Inokuma H, Raoult D, Brouqui P (2000) Detection of *Ehrlichia platys* DNA in Brown Dog Ticks (*Rhipicephalus sanguineus*) in Okinawa Island, Japan. J Clin Microbiol 38: 4219–4221.

35. Werren JH, Baldo L, Clark ME (2008) *Wolbachia*: master manipulators of invertebrate biology. Nat Rev Microbiol 6: 741–751. doi:10.1038/nrmicro1969.

36. Menchaca AC, Visi DK, Strey OF, Teel PD, Kalinowski K, et al. (2013) Preliminary assessment of microbiome changes following blood-feeding and survivorship in the *Amblyomma americanum* nymph-to-adult transition using semiconductor sequencing. PLoS ONE 8: e67129. doi:10.1371/journal.pone.0067129.

37. Kneip C, Lockhart P, Voss C, Maier U-G (2007) Nitrogen fixation in eukaryotes - new models for symbiosis. BMC Evol Biol 7: 55. doi:10.1186/1471-2148-7-55.

38. Van Borm S, Buschinger A, Boomsma JJ, Billen J (2002) *Tetraponera* ants have gut symbionts related to nitrogen-fixing root-nodule bacteria. Proc R Soc B Biol Sci 269: 2023–2027. doi:10.1098/rspb.2002.2101.

39. Kautz S, Rubin BER, Russell JA, Moreau CS (2013) Surveying the microbiome of ants: comparing 454 pyrosequencing with traditional methods to uncover bacterial diversity. Appl Environ Microbiol 79: 525–534. doi:10.1128/AEM.03107-12.

40. Hawlena H, Rynkiewicz E, Toh E, Alfred A, Durden LA, et al. (2013) The arthropod, but not the vertebrate host or its environment, dictates bacterial community composition of fleas and ticks. ISME J 7: 221–223. doi:10.1038/ismej.2012.71.

41. Salles JF, De Souza FA, van Elsas JD (2002) Molecular method to assess the diversity of *Burkholderia* species in environmental samples. Appl Environ Microbiol 68: 1595–1603.

42. Compant S, Nowak J, Coenye T, Clément C, Ait Barka E (2008) Diversity and occurrence of *Burkholderia* spp. in the natural environment. FEMS Microbiol Rev 32: 607–626. doi:10.1111/j.1574-6976.2008.00113.x.

43. Kikuchi Y, Hayatsu M, Hosokawa T, Nagayama A, Tago K, et al. (2012) Symbiont-mediated insecticide resistance. Proc Natl Acad Sci epub online ahead of print: 1–5. doi:10.1073/pnas.1200231109.

44. Valvano MA, Keith KE, Cardona ST (2005) Survival and persistence of opportunistic *Burkholderia* species in host cells. Curr Opin Microbiol 8: 99–105. doi:10.1016/j.mib.2004.12.002.

45. Navarro-Noya YE, Suárez-Arriaga MC, Rojas-Valdes A, Montoya-Ciriaco NM, Gómez-Acata S, et al. (2013) Pyrosequencing analysis of the bacterial community in drinking water wells. Microb Ecol 66: 19–29. doi:10.1007/s00248-013-0222-3.

46. Milford AD, Achenbach LA, Jung DO, Madigan MT (2000) *Rhodobaca bogoriensis* gen. nov. and sp. nov., an alkaliphilic purple nonsulfur bacterium from African Rift Valley soda lakes. Arch Microbiol 174: 18–27. doi:10.1007/s002030000166.

47. Brown SP, Jumpponen A (2013) Contrasting primary successional trajectories of fungi and bacteria in retreating glacier soils. Mol Ecol epub ahead of print: 1–17. doi:10.1111/mec.12487.

48. Neilson JW, Quade J, Ortiz M, Nelson WM, Legatzki A, et al. (2012) Life at the hyperarid margin: novel bacterial diversity in arid soils of the Atacama Desert, Chile. Extremophiles 16: 553–566. doi:10.1007/s00792-012-0454-z.

49. Ventura M, Canchaya C, Tauch A, Chandra G, Fitzgerald GF, et al. (2007) Genomics of Actinobacteria: tracing the evolutionary history of an ancient phylum. Microbiol Mol Biol Rev 71: 495–548. doi:10.1128/MMBR.00005-07.

50. Eller G, Frenzel P (2001) Changes in activity and community structure of methane-oxidizing bacteria over the growth period of rice. Appl Environ Microbiol 67: 2395–2403. doi:10.1128/AEM.67.6.2395-2403.2001.

51. Anesti V, McDonald IR, Ramaswamy M, Wade WG, Kelly DP, et al. (2005) Isolation and molecular detection of methylotrophic bacteria occurring in the human mouth. Environ Microbiol 7: 1227–1238. doi:10.1111/j.1462-2920.2005.00805.x.

52. Anesti V, Vohra J, Goonetilleka S, McDonald IR, Sträubler B, et al. (2004) Molecular detection and isolation of facultatively methylotrophic bacteria, including *Methylobacterium podarium* sp. nov., from the human foot microflora. Environ Microbiol 6: 820–830. doi:10.1111/j.1462-2920.2004.00623.x.

53. Lai C-C, Cheng A, Liu W-L, Tan C-K, Huang Y-T, et al. (2011) Infections caused by unusual *Methylobacterium* species. J Clin Microbiol 49: 3329–3331. doi:10.1128/JCM.01241-11.

54. Terenius O, Lindh JM, Eriksson-Gonzales K, Bussière L, Laugen AT, et al. (2012) Midgut bacterial dynamics in *Aedes aegypti*. FEMS Microbiol Ecol 80: 556–565. doi:10.1111/j.1574-6941.2012.01317.x.

55. Aylward FO, McDonald BR, Adams SM, Valenzuela A, Schmidt RA, et al. (2013) Comparison of 26 Sphingomonad genomes reveals diverse environmental adaptations and biodegradative capabilities. Appl Environ Microbiol 79: 3724–3733. doi:10.1128/AEM.00518-13.

56. Gronesova P, Ficova M, Mizakova A, Kabat P, Trnka A, et al. (2008) Prevalence of avian influenza viruses, *Borrelia garinii*, *Mycobacterium avium*, and *Mycobacterium avium* subsp. *paratuberculosis* in waterfowl and terrestrial birds in Slovakia, 2006. Avian Pathol 37: 537–543. doi:10.1080/03079450802356953.
